# Guiding organisational decision-making about COVID-19 asymptomatic testing in workplaces: mixed-method study to inform an ethical framework

**DOI:** 10.1186/s12889-022-13993-1

**Published:** 2022-09-15

**Authors:** Jan W. van der Scheer, Akbar Ansari, Meredith McLaughlin, Caitríona Cox, Kathleen Liddell, Jenni Burt, Jenny George, Rebecca Kenny, Ruth Cousens, Brandi Leach, James McGowan, Katherine Morley, Janet Willars, Mary Dixon-Woods

**Affiliations:** 1 THIS Institute, University of Cambridge, Cambridge Biomedical Campus, Clifford Allbutt Building, Cambridge, CB2 0AH UK; 2 Homerton College, Hills Rd, Cambridge, CB2 8PH UK; 3 Faculty of Law, The David Williams Building, 10 West Rd, Cambridge, CB3 9DZ UK; 4 RAND Europe, Westbrook Centre/Milton Rd, Cambridge, CB4 1YG UK; 5 Department of Health Sciences, University of Leicester, George Davies Centre, University Road, Leicester, LE1 7RH UK

**Keywords:** COVID-19, Bioethics, Workplaces, Testing, Qualitative, Survey, Mixed-method

## Abstract

**Background:**

Workplace programmes to test staff for asymptomatic COVID-19 infection have become common, but raise a number of ethical challenges. In this article, we report the findings of a consultation that informed the development of an ethical framework for organisational decision-making about such programmes.

**Methods:**

We conducted a mixed-method consultation – a survey and semi-structured interviews during November–December 2020 in a UK case study organisation that had introduced asymptomatic testing for all staff working on-site in its buildings. Analysis of closed-ended survey data was conducted descriptively. An analysis approach based on the Framework Method was used for the open-ended survey responses and interview data. The analyses were then integrated to facilitate systematic analysis across themes. Inferences were based on the integrated findings and combined with other inputs (literature review, ethical analysis, legal and public health guidance, expert discussions) to develop an ethical framework.

**Results:**

The consultation involved 61 staff members from the case study organisation (50 survey respondents and 11 interview participants). There was strong support for the asymptomatic testing programme: 90% of the survey respondents viewed it as helpful or very helpful. Open-ended survey responses and interviews gave insight into participants’ concerns, including those relating to goal drift, risk of false negatives, and potential negative impacts for household members and people whose roles lacked contractual and financial stability. Integration of the consultation findings and the other inputs identified the importance of a whole-system approach with appropriate support for the key control measure of isolation following positive tests. The need to build trust in the testing programme, for example through effective communication from leaders, was also emphasised.

**Conclusions:**

The consultation, together with other inputs, informed an ethical framework intended to support employers. The framework may support organisational decision-making in areas ranging from design and operation of the programme through to choices about participation. The framework is likely to benefit from further consultation and refinement in new settings.

**Supplementary Information:**

The online version contains supplementary material available at 10.1186/s12889-022-13993-1.

## Background

Asymptomatic testing for COVID-19 for staff in workplaces has become a common response to the pandemic [1–5], based on the principle that early detection of positive cases can, through isolation of individuals and their close contacts, disrupt transmission and reduce risk [6–12]. COVID-19 asymptomatic testing programmes have, however, attracted considerable controversy, for example regarding their efficacy, possible unintended negative consequences, and issues around equity and privacy [6, 10, 13–22]. Many debates so far have concerned large-scale mass testing programmes, such as in public health districts or higher education settings [23–28]. Distinctive issues are raised by organisations choosing to operate workplace asymptomatic testing programmes for their staff [1, 29–32].

These issues arise in a context where workplace health interventions more generally have been a growing focus of tension and debate [33], for example regarding impacts on privacy, risks of discrimination, compromise of employee autonomy and unwarranted employer paternalism [33–36]. A key ethical debate concerns the extent to which the health of an employee is a private matter over which the individual has full autonomy, or whether there are overriding principles – such as an employer’s legal or ethical duty of care to the entire body of employees, clients, and wider society [32–34, 36]. These debates have, for example, been prominent in relation to programmes that test employees for substances that might impair performance in safety–critical industries [37–39], or programmes that involve testing or vaccination for specific infectious diseases, e.g. influenza [40] and hepatitis C [41, 42]. Another set of concerns focuses on the potential for undue influence on consent to workplace health interventions, given employees’ dependent relationship with their employers [36].

These kinds of debates about workplace health interventions are complicated by the acute public health emergency posed by the COVID-19 pandemic [15, 43, 44]. Balancing the rights of individuals, groups and organisations with questions of collective good is not straightforward in the context of a potentially serious infectious disease with a two week infectious period and relatively high levels of asymptomatic infection. The UK government and some advisory bodies have issued guidance on mass asymptomatic testing for COVID-19 during waves of the pandemic (e.g. [17, 45, 46]). Some general guidance for workplace testing has also been issued in various countries (e.g. [1, 4, 5, 29, 30, 47]), but few resources have focused specifically on ethical issues in workplace settings [32].

This is problematic because workplaces considering a COVID-19 asymptomatic testing programme have to grapple with a set of inter-related responsibilities that may not sit easily together, such as the responsibility to provide a safe workplace and to respect individual choices and personal privacy [5, 30]. They generally must also develop policies to swiftly notify public health agencies of positive test results [4, 5, 31], process personal data in fair, lawful and transparent ways [30, 31], and ensure that any testing regime is appropriately certified and compliant with regulatory requirements for health interventions, medical devices, in vitro diagnostics and equality [31, 48–52]. Further, decisions about choice of testing regime and likely effectiveness may need to be made in conditions of great uncertainty, rapidly evolving science and technology developments, and shifting policy contexts [53]. In such circumstances, the need to identify and address ethical issues using participatory approaches with involvement from different stakeholders has been repeatedly emphasised [54–57]. However, this need has not yet been addressed in the context of asymptomatic COVID-19 workplace testing programmes.

In this article, we address this void. We report the findings of a consultation with staff in a case study organisation that had introduced asymptomatic testing for all staff working on-site in its buildings. The findings were combined with other inputs (i.e. literature review, ethical, legal and public health guidance, expert discussions) to inform the development of a framework to guide organisational decision-making about testing staff for asymptomatic COVID-19 infections.

## Methods

We used a multi-stage iterative process to develop an ethical framework for asymptomatic COVID-19 testing in workplaces, using methods very similar to those we have previously reported in relation to an ethical framework for COVID-19 testing in higher education institutions [23], including:

1. development of an initial provisional framework to inform the topics and analysis of the consultation;
2. consulting staff at a case study organisation using a survey and semi-structured interviews;
3. analysing and integrating the survey and interview data; and
4. drawing inferences from the integrated consultation findings and other inputs (e.g. literature review, ethical analysis, public health guidance, and the authors’ expertise) to devise an ethical framework.

### Provisional framework

A provisional framework informed the topics of the consultation’s survey and semi-structured interview guide. It also informed initial analysis and inferences drawn from the consultation’s findings. Development of the initial provisional ethical framework was informed by three primary resources. First, we drew on an ethical framework previously developed for COVID-19 testing for NHS workers [58]. Second, we conducted an informal literature review and analysis of ethical, legal and practical issues potentially relevant for asymptomatic workplace testing, such as public health ethics (e.g. [32, 54, 56, 59–62]), ethics of workplace health interventions (e.g. [33–36]), and official governmental guidance on asymptomatic testing. And third, we drew on author team expertise in law, ethics, social science, anthropology, public health and healthcare improvement.

### Case study mixed-method consultation

We used a mixed-method approach (a survey and semi-structured interviews) to consult employees at a case study organisation that had introduced asymptomatic testing for all staff working on-site in its buildings. The organisation’s workforce was mostly desk-based, but a substantial minority of staff had public-facing and client-facing roles (including hospitality, retail and reception). The testing programme involved nasal self-swabbing by staff at home (before travelling to work), followed by a polymerase chain reaction (PCR) analysis of samples conducted by a third-party laboratory. The programme’s policy was that testing was mandatory for anyone needing or wishing to work onsite.

Our consultation took place in November–December 2020, during a period when the programme was already implemented on a small scale for members of the workforce who were required to be on-site during the early months of the pandemic. The programme was planned to be rolled out to the remaining staff (who had been working from home) as part of the more general return to buildings.

We used a mix of convenience and purposive sampling [63, 64], with the aim of achieving diversity of perspectives and socio-demographic characteristics. Participants were initially recruited through messages on the organisation’s intranet (convenience sampling). A second wave of recruitment was employed using targeted email invitations to individuals with various roles in the organisation (purposive sampling). Participants visited a webpage with further information and then registered for the consultation on Thiscovery (https://thiscovery.org/about), an online research and development platform created and developed by The Healthcare Improvement Studies (THIS) Institute at the University of Cambridge. Written consent was provided by all participants prior to the interview or survey. A range of socio-demographic data was collected (e.g. age, gender, ethnicity, disability, job role, caring responsibilities), but, to preserve confidentiality, are not reported in detail here.

Participants had the option of participating either in an online survey or a semi-structured interview. The survey and interview prompt guide (see Additional files 1 and 2) were based on the provisional framework and refined through piloting with staff of the organisation. The survey was hosted on Thiscovery, and took approximately 15 to 20 min to complete. It included a mix of five-point Likert-scale and open-ended questions. The interviews were conducted with an experienced interviewer and took place online using video software. They lasted 30 to 90 min. The interviews were transcribed verbatim. All data were anonymised for identifying information about individuals or organisations.

### Mixed-method analysis

The survey and interview data were initially separately analysed; integration of the data took place at the interpretation stage of analysis [65–67].

We analysed closed-ended survey data (i.e. five-point Likert-scale survey question responses) using descriptive statistics and diverging stacked bar charts [68] implemented in the R statistical software as outlined elsewhere [69]. Open-ended survey responses and interview data were analysed using an approach broadly based on the Framework Method [70]. This approach enabled multiple analysts to examine the data, looking for commonalities and divergences in the data by comparing views of participants. After familiarisation with the interview data, two analysts (JG and BL) independently coded the first three transcripts deductively using pre-defined codes based on the provisional ethical framework. Several authors (JWvdS, AA, CC, MM, MDW) then examined the work of JG and BL to agree on a coding framework to apply to all transcripts. The coding framework was applied to all transcripts to produce a matrix, and a summary was written for each code. We analysed the open-ended survey data similarly to the interviews, using a separate matrix and coding summaries for these data.

For data integration at the interpretation stage of analysis, the authorial team discussed patterns arising across the analyses, starting with arraying the three types of data together to facilitate systematic analysis across themes. The discussions then involved an iterative process to review the data to generate themes, including the consideration of convergence and divergence between data sources. In the interpretation stage, equal priority was given to the three different types of data. We did not undertake a formal test for theoretical saturation; instead, we used the principle of information power to confirm that we had captured a sufficient range and depth of views [71].

### Development of the final framework

We sought to produce a final framework with themes based on wider ethical thinking, the literature, available guidance and professional expertise, while also taking account of the consultation findings. As part of a process of reasoned and deliberative justification [72], we engaged in multiple rounds of iterative analysis and discussion across several weeks. This analysis included interpretation of the consultation findings by the authorial team and iterative synthesis with their professional expertise and knowledge of the literature across ethics, public health, law, anthropology, social science, and healthcare improvement. To conduct ethical analysis, we reviewed existing theories, principles and frameworks in relevant areas, with attention to the potential ethical challenges relevant to COVID-19 testing programmes and infectious diseases outbreaks (e.g. [32, 54, 56, 58–62, 73]). We also examined applicable legal and regulatory requirements related to testing and information governance (e.g. [1, 5, 17, 29–31, 45–52, 74]).

## Results

In all, 61 staff at the case study organisation took part in the consultation: 50 in the survey and 11 in an interview, representing approximately 10% of the organisation’s workforce. Diversity of demographics and job roles of the participant groups largely matched those of the organisation itself (when compared to available human resource data), with the exception that casual or manual workers were largely absent.

The socio-demographic data showed that most participants (*n* = 48) were aged 20 to 45 years old; the others were aged 46 to 60 years (*n* = 7) or did not report their age (*n* = 6). About two-thirds of participants identified as a woman, the others identified as a man or with a different gender orientation. White ethnicity was reported by 46 participants; the others reported Asian, African or mixed ethnicities. Seventeen participants reported having caring responsibilities, and four participants reported having a disability; 48/61 had a permanent contract; 53/61 were in full-time positions. Around half had a junior professional role (*n* = 33), a smaller proportion an intermediate managerial role (*n* = 24), and a small number had a higher managerial role (*n* = 3).

Our analysis resulted in eight themes under which we organised the consultation data as part of the framework: 1) design and operation of the programme; 2) goals of the programme; 3) properties of the test(s); 4) enabling isolation; 5) choices regarding participation; 6) benefits, harms and their distribution, including opportunity costs; 7) privacy, confidentiality and data-sharing; 8) communication. For each theme below, we first provide the main recommendation as part of the framework (Table 1). We then provide the reasoning behind the framework’s theme, including reference to consultation data and other inputs that informed the framework’s set of recommendations. Reference to consultation data is enriched with survey graphs and illustrative data from the open-ended survey responses and interviews (Additional file 3). Reasoning and inferences for some themes drew mostly on findings from the consultation; other themes were more heavily influenced by wider ethical and legal reasoning, public health literature and guidance, and multidisciplinary professional expertise grounded in the literature.

**Table 1:** Ethical framework for asymptomatic COVID-19 testing of staff in workplaces

Ethical consideration	Recommendations	How might these recommendations be put into practice?
**Design and operation of the programme**	**Start by making an assessment of whether a programme is the right choice for your organisation**, taking account of the available evidence, current pandemic conditions, and your own resources and capabilities. **Ensure that all other infection control measures are in place** both before making a decision about introducing a testing programme and after the decision. A testing programme should be seen as one element in a multi-modal strategy. **Recognise that a testing programme requires a whole-system approach**. Testing on its own is not enough. There must also be confidence that the key control measure (isolation of confirmed cases and close contacts) can be achieved. **Sign up in full to delivering on all of the components necessary for the testing programme**, ensuring that adequate resources, capabilities and quality assurance mechanisms are in place. **Set up a governance structure capable of dealing with all aspects of the programme**, including clear decision-making, operational oversight, quality assurance, communication, and facilities for consultation and ethical advice. **Plan for a range of scenarios and ensure that contingencies are in place.** **Consider whether there may or may not be a duty of care to** operate a testing programme if current evidence points in favour of the benefits of a testing programme outweighing any downsides and the organisation is reasonably able to offer a programme.	**Some hypothetical examples** • An organisation where much of the workforce is required to be in the workplace, and with many staff in public-facing roles, makes a full assessment of whether an asymptomatic COVID-19 testing programme could reduce risk of infection and contribute to improved safety for colleagues and clients. It considers how likely it is that it can reasonably achieve these aims given current pandemic conditions, the available technologies, and its ability to deliver a programme in full. • An organisation identifies all the components that need to be in place for the programme to work well. It makes an assessment of the costs, opportunity costs (e.g. things it can’t do because it is doing the programme), risks and possible unwanted consequences of the programme. It considers the available evidence on testing programmes, current pandemic conditions, its own business needs, its ability to commit resources, and the likely willingness and ability of the workforce to participate. It is confident that isolation of cases that test positive can be achieved. It concludes that running a testing programme is a justifiable addition to its multi-modal approach to ensuring a safer workplace. • A different organisation, having reviewed the resources required to deliver all elements of a testing programme effectively, determines that it lacks the financial and logistical capabilities to run a sufficiently quality-assured and effective programme. It therefore decides not to proceed with a testing programme. • An organisation that has decided to proceed with a testing programme creates a sound governance structure. It identifies key responsibilities and accountabilities in the programme team, a clear decision-making structure, and a resources plan. The programme team has operational strengths and sound understanding of the science and ethics of testing, and is aware of the public health and legal responsibilities associated with testing. The team designs a holistic, end-to-end programme. The organisation designs and implements appropriate quality assurance mechanisms. It is alert to public health guidance and government policy as it changes over time, and makes appropriate adjustments to its programme. An organisation running a testing programme models a number of scenarios and puts plans in place to cope with them, for example to address business continuity in situations where large numbers of staff test positive. • An organisation that has decided to run a testing programme conceptualises it as a duty of care to staff and clients, and as a benefit to staff and the community. It presents the programme to its stakeholders in this way, with the aim of engendering trust, solidarity and a sense of mutual responsibility. The organisation also identifies any factors which might undermine trust (e.g. treating some groups unfairly, communication failures, poor logistics, lack of support for isolation) and takes steps to address these issues.
**Goals of the testing programme**	**Ensure that the goals of the programme are well-defined and have a clear rationale** based on disrupting viral transmission through early control measures, especially isolation of positive cases and their contacts. If there are multiple programme goals, they should be acknowledged explicitly. **The goals should be clearly explained to staff.** **Goals should be realistically attainable**, based on current understanding of the epidemiology of COVID-19, the properties of the selected testing regime, and the available resources and measures for managing risk. **Specify criteria to judge the effectiveness** of the programme in reaching its goals. **Keep the programme goals under active review**, mindful that they may evolve over time. Goal drift (uses of the programme for purposes not specified) should be avoided.	**Some hypothetical examples** • An organisation defines the goals of its asymptomatic testing programme as improved workplace safety for all colleagues and fulfilling duties of care to client groups. Secondary goals include delivering broader public health benefits and providing reassurance for staff and clients. The organisation is clear about the underlying rationale for why these goals might be achievable: detecting asymptomatic infection enables isolation of positive cases and close contacts, and therefore reduces onward transmission. It also acknowledges the current uncertainties associated with demonstrating whether the programme can deliver on its goals. • An organisation communicates that the wellbeing and safety of staff and clients is the primary motivation for the programme. It clarifies the scope of the programme, explaining that the programme is intended to identify positive cases who can then isolate to disrupt viral transmission. Following feedback from staff, it reaffirms that it is not using negative tests as a way of forcing people to come to the workplace when they could reasonably work from home for the present. • An organisation specifies that it will judge the effectiveness of the programme using criteria relating to: participation rates, positivity rates (percentage of people tested who are positive), outbreaks, cost-effectiveness, and staff satisfaction (measured by surveys). It acknowledges influences outside its own scope of control, such as community prevalence. It monitors effectiveness over time. It keeps its programme under review as conditions, technology, policy, guidance, scientific understanding, and staff views evolve. It clearly signals any changes to all stakeholders. • An organisation plans regular reviews to check that the purposes being served by the programme are still valid and relevant to need, and to ensure that goal drift has not occurred (e.g. using data from the programme as a means of surveillance of productivity or attendance).
**Properties of the test(s) selected for the programme**	**Be alert to the properties of test selected for the programme and the implications of these**. Test properties, such as sensitivity and specificity, may vary considerably depending on the test used and the setting (including whether or not swabbing is self-administered). **Ensure that the methods of obtaining the sample (e.g. swabbing) satisfy criteria of tolerability and acceptability** (e.g. not inducing excessive discomfort, pain, or anxiety). **Consider the probabilities of false negative and false positive test results associated with the chosen testing regime, and identify and mitigate the possible associated risks and harms.** **Emphasise prominently and consistently the importance of continuing to observe guidance on masking, social distancing, hygiene and ventilation in the event of negative tests.** **Clearly communicate what should happen in response to a positive test**, including any opportunity for confirmatory testing and the support available. **Acknowledge the implications of false negatives and false positives** in communications about the programme. **Be alert and responsive to changes in evidence surrounding testing technologies, and be aware of current government guidance.** **Be mindful of relevant legal and regulatory requirements relevant to testing**. Amongst other things, you should consider the current authorisation and certification of the devices being used for testing, and any requirements for laboratories being used (whether in-house or under contract) to be certified.	**Some hypothetical examples** • An organisation makes a full assessment of the available testing options. It considers validity and reliability, convenience and speed of administration and test result, tolerability of the test for staff, costs, logistical burden and test certification. • An organisation takes steps to ensure that swabbing is done correctly. It reassures staff that there is only mild physical discomfort associated with a nasal swab. It posts a video showing the correct technique for self-swabbing on its website, with voiceovers led by staff themselves, and emphasises that support is available should the test outcome trigger self-isolation. The organisation acknowledges the possibility that some people might experience anxiety about taking the test, about the results of the test, or about the impacts that test results could have for people and their households. It ensures that these concerns are not trivialised. It provides opportunities for staff to talk to people who have already had the test to answer any questions. • One organisation, having reviewed the latest government advice and other sources, selects lateral flow testing. It checks that the system has been certified for the purposes for which the organisation is planning to use it. It recognises the limitations of the currently available form of the technology, including the risk that its poor sensitivity could generate high false negative rates (people testing negative for COVID-19 even though they have it). Accordingly, the organisation emphasises in all communications that, for the present, the main goal of the programme is to detect positive cases in asymptomatic individuals. It stresses that the programme does not provide evidence of non-infection, and that negative results should not be used to support relaxation of compliance with social distancing, face coverings, or hygiene rules. • A different organisation also conducts a full assessment of the available testing options. It decides to use PCR testing, and contracts with an external laboratory to provide the facilities needed. It ensures that the lab is certified appropriately for the tests, so it meets the required legal standards. • An organisation reminds its staff of current national guidelines regarding whether it is possible to return to work after close contact with a confirmed case of COVID-19 on the basis of a negative test. • An organisation considers the probability of false positives associated with its chosen testing regime. It notes that in the event of a false positive, a staff member and their close contacts will be erroneously required to isolate. To mitigate this risk, it offers swift confirmatory testing. • An organisation discovers that an unintended consequence of the programme is that those who test negative may engage in more risk-taking behaviour. In response, it explicitly communicates that a negative test result means an individual “has tested negative, but could still be infected with the virus”. It informs staff that the asymptomatic testing programme is “one tool in a multi-component risk reduction strategy”. It increases its communication about distancing, face-covering, hygiene, and other infection control measures.
**Enabling isolation**	**Ensure that adequate support is in place for isolation after a positive test, both at individual and household level**. Programme effectiveness in breaking chains of transmission depends on individuals and their close contacts isolating after a positive test. **Be clear in communication about both the requirement for isolation and the available support**. A dialogue should take place to ensure that those isolating feel heard, and the experiences should be used to guide the refinement of support systems.	**Some hypothetical examples** • An organisation emphasises to staff that they must self-isolate if they test positive or have had close contact with a positive case. It assesses what is needed to support this. It is aware of worries and concerns staff may have when self-isolating (e.g. anxiety, loneliness, limited access to food shopping and exercise, difficulties related to caring responsibilities, and impact on household members who may also have to isolate as close contacts). The organisation makes sure that isolating staff receive paid leave and practical support where needed. It makes mental health support resources available. Using a clear process, it offers additional financial assistance for staff who might otherwise struggle (e.g. because they or their household members are on zero hours contracts). • An organisation communicates clearly with its staff about what to do if they receive a positive result, what support is available if they must isolate and how they can access it. Communication is intended to address concerns and alleviate anxiety around the possibility of having to isolate; the organisation is attentive to feedback from staff about the experience of isolating, and demonstrates that it is acting on it to reduce the burdens of isolation.
**Choices regarding participation in testing programmes**	**Make a reasoned decision about how far staff participation in the programme should be voluntary or mandatory, with explicit attention to ethical principles, relevant laws and the nature of the employee/employer relationship**. Some considerations include (but are not limited to) the priority to be given to individual choice, the extent to which the effectiveness of the programme depends on full participation, and the possibility of creating unfairness (for example if choice is more easily exercised by more senior groups while increasing risk for front-line groups or by undermining the efforts of those who do participate). **If the decision is taken to mandate programme participation, explain the rationale clearly**. The reasons for any restrictions on individual choice should be clearly articulated, and any opportunities for individual exceptions to be made (and the criteria that might apply) should be explained. Where a decision is taken to mandate a testing programme, **communication should be sensitive to the potential for industrial relations conflict (e.g. with trade unions or staff representatives), hostility, loss of trust, and possible poorer compliance with the programme.** **Consider the use of any incentives carefully**. If used, they should be small and presented as tokens of appreciation rather than stimulation to take part. In general, **avoid penalties (or measures that have the appearance of penalties)**, because they have the potential to impact on trust, undermine solidarity, cause resentment, or lead to a burden of complaints to be managed.	**Some hypothetical examples** • An organisation offers individual staff the choice of whether to participate in the testing programme or not, supported by clear communication surrounding the potential benefits of the programme (including the shared benefits of health protection and community solidarity). Following complaints from some staff about having to share workspace with untested colleagues, an organisation decides to reiterate the principle that participation in testing is voluntary at an individual level, but makes it clear that it strongly encourages staff to participate for the safety of others unless they have good reasons not to participate. It commits to keeping the policy under review if new evidence emerges. • A different organisation makes it clear that its expectation is that all staff will participate in the programme, but it provides a formal process for individuals to apply to be exempted from participation. Its communication focuses on the need for solidarity and collegiality in reducing viral transmission, the shared benefits of reduced risk, and averting unfairness. It reiterates its commitment to equality, diversity and inclusion. • Following consultation with staff representatives, another organisation decides to mandate the programme for all staff who work on its premises. It assesses that making it voluntary could lead to the programme goals being undermined (reduced ability to disrupt transmission through identification and isolation of positive cases), could potentially lead to unfairness across different staff groups, and could be a source of tension between colleagues. The organisation explains that it will consider carefully any concerns as part of its programme’s governance mechanisms. It provides a formal process through which individuals can apply to be exempted from participation. • An organisation, following consultation, concludes that offering small incentives (such as a free coffee for taking part) to encourage participation would be acceptable. It monitors for any unintended consequences, such as the perception that offering either excessively trivial or unduly large rewards could undermine a programme built on community spirit.
**Benefits, harms and their distribution, including opportunity costs**	**Consider the possible benefits, risks and harms of the programme, including issues of equality, diversity and inclusion**. Put appropriate mitigations in place where possible. **Identify any staff groups who might be disproportionately affected by the testing programme and seek to mitigate the effects.** **Consider the trade-offs and opportunity costs (e.g. things that could not be done because of running the programme)**, the range and nature of uncertainties, and the need to balance values that may be difficult to reconcile (e.g. individual liberty and collective benefit). **Be able to give an account of the reasonableness of your organisation’s decision-making process and its decisions**. People may reasonably disagree about how an organisation should weigh up possible benefits, risks and harms, and the different views on these questions, so be prepared to explain how the decisions have been made.	**Some hypothetical examples** • An organisation considers the possible benefits and harms of the programme and explicitly considers equality, diversity and inclusion in their distribution. It identifies that those on low incomes or pre-existing mental health conditions may be disproportionately disadvantaged by isolation. It also identifies that some appear to be experiencing stigma and discrimination in relation to non-participation in the programme. The organisation puts appropriate measures in place to address these challenges, including reminders that some people have legitimate reasons not to participate, a hardship fund for staff facing particular difficulties, reduced productivity expectations, and paid leave. • An organisation examines costs and benefits of the programme. It considers alternative uses to which the resources might be put, and decides that the programme is a justified expenditure for now because of its potential to reduce transmission of COVID-19 for staff, clients and the community, while also facilitating business continuity. The organisation reviews this assessment at key points, for example when costs increase or benefits appear to decrease. • An organisation, through its governance mechanisms, makes clear and explicit the rationale underlying its decisions and acknowledges that not everyone will agree with all of them. It uses consultation where appropriate to engage with diverse views and ensure that they have been taken into account.
**Privacy, confidentiality and data-sharing**	**Design and operate sound systems for information governance, recognising that information about test participation and test results should be handled with full respect for principles of data protection and confidentiality**. Data should be processed fairly, lawfully, transparently and securely and in accordance with data protection principles specified in General Data Protection Regulation (GDPR). Confidential health information can only be used with the individual’s consent, or where it is necessary and proportionate to protect public safety and the health and safety of other people. **Consider whether a data protection impact assessment for the programme is needed.** **Ensure that individuals processing programme data are properly trained.** **Communicate clearly about who will be informed in the event of a positive test result**, with clear justification for any sharing of health data. **Communication should make clear that public health authorities may be notified of positive test results, thereby activating contact tracing mechanisms.** **If you conduct contact tracing on-site (e.g. before the public health authorities take over), you have a duty to inform contacts that they have been exposed** (which may result in deductive disclosure, as colleagues may guess the identity of the infected individual). **Be mindful of relevant legal and ethical requirements of any use of data for research purposes.**	**Some hypothetical examples** • A large organisation conducts a data protection impact assessment for its testing programme. It establishes clear workflows for handling data at every stage, and ensuring full compliance with the principles specified in the General Data Protection Regulation. Data is pseudonymised or anonymised where possible. The organisation establishes a secure system where test data are stored in a protected space with access limited to dedicated testing programme managers. It trains individuals handling data, ensuring that they are aware that data concerning health is confidential: it can only be used with the individual’s consent or where it is necessary and proportionate to protect public safety and the health and safety of other people. The organisation issues a privacy notice with information on sharing of personal data. • An organisation develops a clear workflow for notifying public health authorities of confirmed positive tests, making it clear to staff that this will activate contact tracing. • An organisation makes sure test results from the laboratory it has commissioned for its programme is feeding the results through to its country’s established public health channels. • An organisation specifies that those who are deemed close contacts by virtue of sharing the same environment in the workplace will be notified that there has been a positive test, but not the name of the individual who has tested positive. It acknowledges that people may be able to work out who has tested positive, but also says it is taking all possible steps to protect confidentiality so, for example, will not confirm “guesses”. • An organisation seeks the consent of staff for their test results to be used for scientific purposes, and obtains the proper approvals to enable use for research purposes. Those who decline consent for this purpose are reassured that there will be no detriment to them.
**Communication**	**Prioritise high quality communication about the programme.** **Provide clear and understandable information** about all aspects of the programme in a range of accessibility-friendly formats. **Be mindful of need for programme trustworthiness, ensuring that communication is respectful and well-judged and addresses issues of equality, diversity and inclusion appropriately.** **Provide a way for staff to provide feedback and to raise concerns about the programme**, for example anonymous reporting through a programme website, and ensure there is a system for acknowledging and addressing the issues raised.	**Some hypothetical examples** • An organisation develops a sound communications plan for the programme. It understands that staff need clear information about a range of topics surrounding a testing programme, but is also attentive to the need to avoid information overload. It designs concise brochures, posters, website and emails, using appropriate language and images that are accessibility-compliant and respectful of diversity. It offers clear signposting to more detailed information. It emphasises consistently the importance of isolation in response to a positive test. In all communication, the organisation strongly emphasises the need for all measures for reducing infection risk, stressing that testing is just one of these. • An organisation identifies email is an efficient and acceptable way of communicating with staff about the programme. It supplements email communication with other resources, including a dedicated and regularly updated programme website. Links are provided to brief videos showing how to administer nasal swabs and other advice about the programme, featuring staff from diverse backgrounds. • An organisation becomes aware of the possibility of stigma linked to testing positive (for example owing to a perception that it reflects reckless or selfish behaviour). In an effort to address this, it emphasises someone can test positive even if they have tried their best to follow guidelines. • An organisation regularly releases statements outlining how it has been collecting feedback from stakeholders on the programme, outlining what changes, if any, it has made in response.

See for supportive resources (e.g. infographic): https://www.thisinstitute.cam.ac.uk/research-articles/covid-19-ethical-framework-for-asymptomatic-testing-of-staff-in-workplaces/

### Design and operation of the programme

#### Main recommendation: Assess whether a testing programme is the right choice for your organisation and whether you can deliver all aspects of it. Ensure you can meet public health and legal duties

The need to deliver, coordinate and quality assure each element of public health programmes [75], including those for COVID-19 testing [6, 17, 76], are well-established principles of public health systems design. A crucial feature of testing is that, on its own, it is not enough to reduce transmission [77–79]; it should be seen as one component of a *whole-system approach* [6, 17, 76, 80, 81]. Testing programmes need to meet public health and regulatory duties (e.g. [1, 5, 17, 29–31, 45–52, 74]). A further key consideration is the possible duty of care an organisation may have to the entire body of staff, clients, and wider society [32–34, 36] to operate a testing programme.

**Fig. 1:**
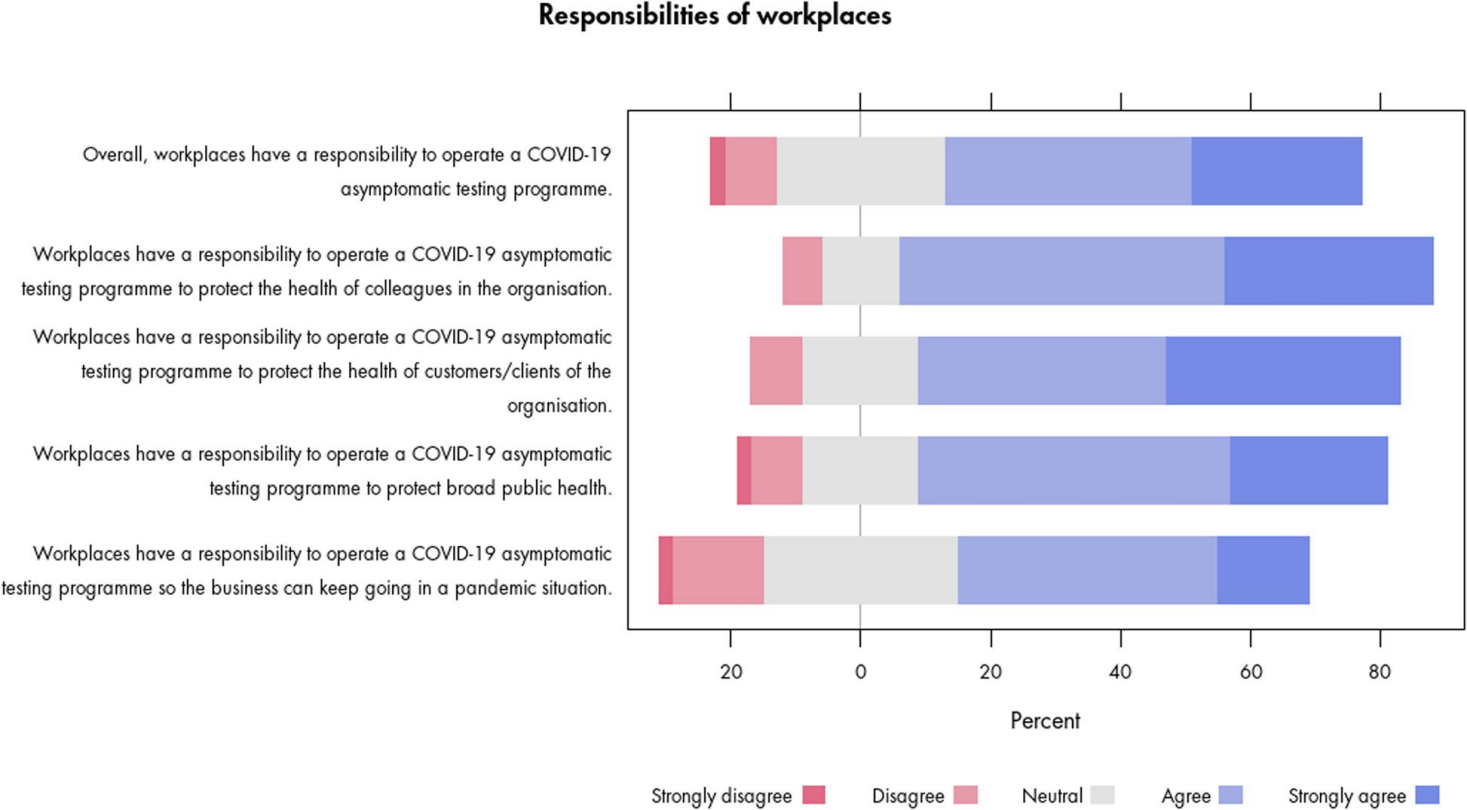
Survey participants’ views on responsibilities of workplaces to operate a testing programme

Most survey respondents (64%) agreed and very few disagreed (10%) that workplaces have a responsibility to operate a COVID-19 asymptomatic testing programme (Fig. 1). While acknowledging the importance of an infection reduction approach that goes beyond testing, the open-ended survey responses and interviews suggested that the nature, size and resources of the organisation matter when considering a responsibility (Additional file 3).

“If a business requires staff to be physically present in the workplace than perhaps the responsibility for asymptomatic covid testing is higher.” (survey)

Interview participants expressed some concerns about whether an asymptomatic programme was the right choice for their organisation (Additional file 3). Many were of the view that the programme was a reasonable intervention for those who wanted or needed to work in the organisation’s buildings, or to ensure business continuity:

“… if it highlights a trend or something, so if a certain team were wiped out by it, then that’s big data to use and the advantage is you can shut down maybe the organisation or part of an organisation very quickly ...” (interview)

Overall, the data and our wider analysis indicate for organisations to assess whether a testing programme is the right choice for them, for example given that is not fully clear how much testing adds beyond other measures (e.g. mask wearing, social distancing) to reduce COVID-19 infections [77, 80, 82–86].

### Goals of the testing programme

#### Main recommendation: Identify the programme goals, explain why they were chosen, tell staff about them, and keep them under review

Goal clarity and legitimacy of goals are important attributes of justifiable and effective public health interventions [55, 59, 60], including COVID-19 testing programmes [17, 32, 79, 87]. One clear goal of a workplace COVID-19 testing programme is to control infection by reducing transmission of the virus in the workplace [17, 47]; another goal might be focused on business continuity [47]. Public health literature and guidance on COVID-19 testing [17, 55, 59, 60, 79], together with the participants’ views, indicate that it is important to acknowledge and communicate all the goals of a workplace COVID-19 testing programme, and make it clear if there are multiple goals.

In our consultation, we found general support for the goal of the programme in reducing infection risk. A large majority of survey participants agreed that workplaces have a responsibility to operate a testing programme to protect the health of colleagues (82%), customers/clients (74%) or broad public health (72%), while very few (10%) disagreed with these proposed responsibilities (Fig. 1). Less support – though still substantial – was expressed for organisations operating a testing programme to ensure business continuity (64% agreed; 15% neutral; 16% disagreed). Virtually all (98%) of the survey participants supported the use of anonymised data from the programme for research purposes. As further illustrated in Additional file 3, some survey participants qualified their views towards organisational responsibilities and goals of a testing programme:

“Is it the responsibility of workplaces to protect broad public health? If that workplace is a hospital or a health provider, maybe, but I don’t know that it applies universally.” (survey)

Other advantages highlighted by the survey participants could be defined as secondary goals of the testing programme: they included reducing anxiety, enhancing feelings of safety, and feeling that employee health matters to the organisation (Additional file 3). Interview participants expressed similar views, supporting the idea that testing could help to reduce risks for colleagues, clients and the wider public, perhaps facilitate business continuity, and provide reassurance to those coming into the buildings (Additional file 3). In addition, interview participants expressed concerns about potential goal confusion or goal drift (Additional file 3), for example using it as a means of forcing people back to buildings when there was no current need and when it might be risky:

“I just don’t think it’s appropriate for workplaces to push people to come back before they’re vaccinated, if there isn’t an impact on their ability to do their jobs, or if there isn’t a significant impact.” (interview)

Our findings highlight the need for clarity about goals and avoidance of goal drift (substituting the original goals with different goals). They also highlight the need to keep the goals under review in line with public health literature arguing that it is pivotal to evaluate the effectiveness of public health interventions [59].

### Properties of the test(s) selected for the programme

#### Main recommendation: Assess the available testing options, considering current evidence and guidance. Acknowledge uncertainty, take action to address risks of the chosen approach, and make sure other infection control measures are maintained

Key ethical requirements for screening programmes include test properties such as sensitivity, specificity, and positive/negative predictive value [88, 89], and the tolerability and acceptability of tests [88–92]. Public health principles further stipulate the need for reflection on the burdens or harms associated with an intervention, including the need for mitigating steps for potential burdens or harm [59]. For a COVID-19 asymptomatic testing programme, harms related to false negative or false positive test results need to be considered and mitigated [14, 19, 26, 93, 94]. Of relevance is reviewing the currently available evidence about which testing method to employ: for example, lateral flow testing and PCR testing have different test properties [95–99]. Organisations need to remain informed about factors such as test properties, convenience and speed of conducting tests and getting results, and how tolerable the test is perceived to be [100, 101]. They also need to remain aware of contemporary government guidance and policy, and be conscious that available technologies and evidence may continue to evolve [1, 53].

**Fig. 2:**
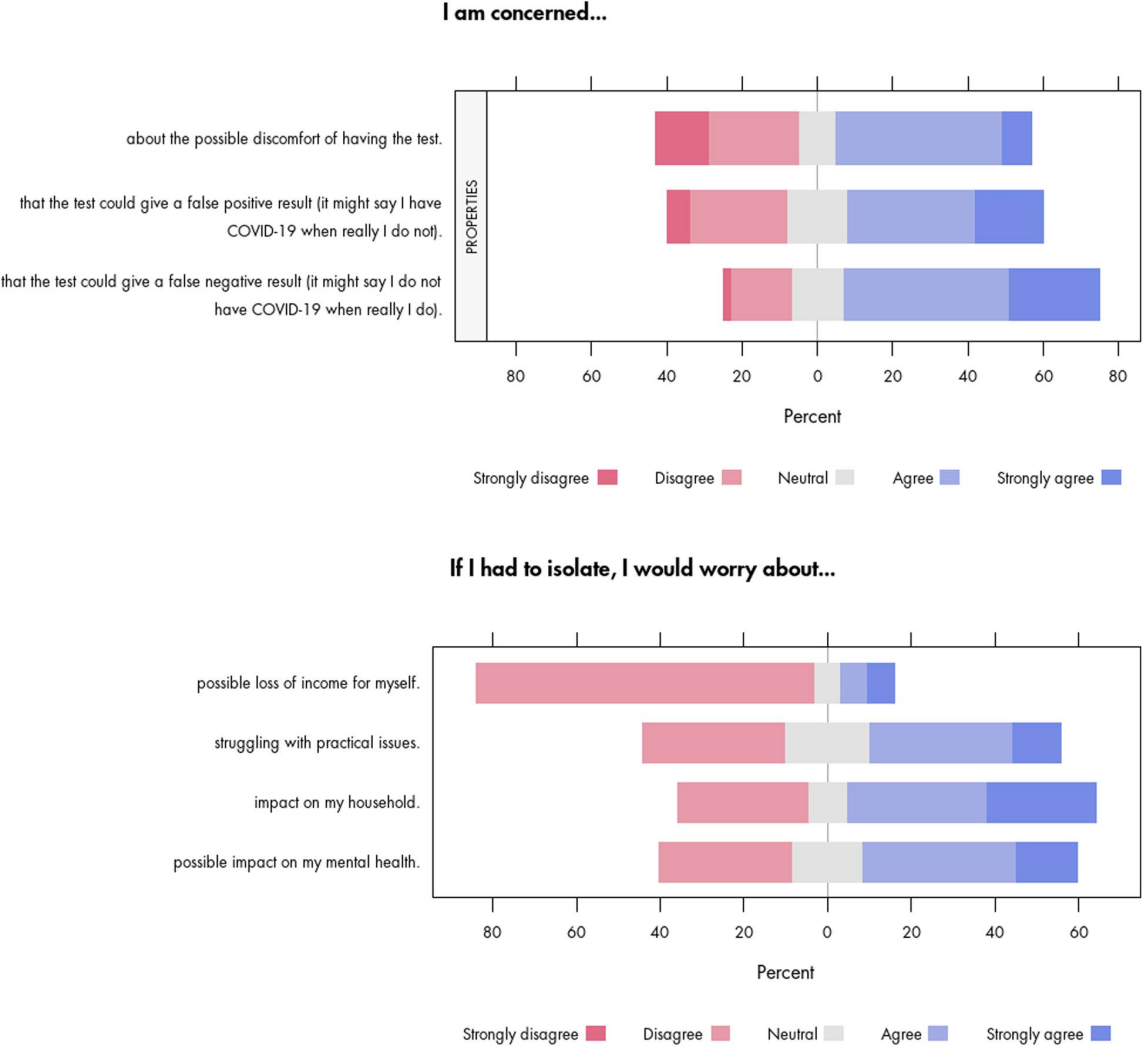
Concerns and worries of survey participants about properties of the test and self-isolation

Concerns over the harms associated with the test itself were evident in our consultation. About two-thirds of the participants (68%) were concerned about the potential for false negative test results, and just over half (52%) were concerned about the potential for false positives (Fig. 2). Similarly, just over half of participants (52%) reported to be concerned about the possible discomfort of having a test (Fig. 2), though of note is that most of the participants at the time of the consultation had not yet undergone workplace testing.

The open-ended survey responses and interviews also offered some insights into concerns about testing, such as it potentially being uncomfortable, uncertainty about time between testing and testing results, and stress over not carrying out the self-swabbing correctly such that it could potentially lead to a false negative result (Additional file 3). Interview participants said they understood that tests are not perfect and would still support people acting on the test results even if a minority of results were incorrect.

“… even if can't be 100 per cent accurate […] I would still be supportive of self-isolating in that situation, even if there's a chance that maybe you don't have it.” (interview)

Some mentioned that if the testing was not sufficiently accurate they would want to have a second test to confirm a result (Additional file 3). Interview participants also wondered about the choice of the type of test (Additional file 3), and suggested that the tests could be seen as a medical procedure with the need for according consent:

“… it needs to be considered a medical procedure because it is essentially. […] anything where people are consenting to a medical procedure then thought needs to be given around the reasons, and being informed about their reasons as well for having or not having it.” (interview)

The literature and our analysis suggest that organisations need to select the test they use carefully [79], and help mitigate staff concerns, uncertainties and anxieties about testing [26, 73, 102]. This can be done by considering current guidance and evidence together with effective communication strategies about uncertainties of the test chosen [103].

### Enabling isolation

#### Main recommendation: Be clear about requirements for isolation. Make sure the right support and communication is in place to support staff and their households to isolate effectively

There has been wide discussion regarding the ethics of isolation and quarantine for communicable diseases, for example in relation to the justification of restrictive measures [104–106]. A recurring theme is that where a restrictive public health intervention is imposed on a population, there is a reciprocal responsibility to ensure that people are provided with the means/support to adhere to it [32, 81, 106–108]. For a COVID-19 testing programme, the identification of people testing positive must be combined with sufficient isolation to effectively stop or reduce virus transmission [80, 109]. The need for support for self-isolation has strongly been emphasised in discourse surrounding COVID-19 testing programmes more generally [29, 79, 81, 109–111]. Preliminary UK evidence suggested sub-optimal compliance with self-isolation, and also indicated that practical support and financial reimbursement could help to address this [112, 113]. In this context, a workplace operating a testing programme might be assumed to have a responsibility to ensure that adequate support is in place to facilitate self-isolation for staff who test positive.

The survey responses in Fig. 2 show that the most frequently reported worries related to self-isolation were about the impact on people’s households (60%), mental health (51%), and practical issues (46%). Although a majority of participants (81%) were not worried about possible loss of income (Fig. 2), some did raise other concerns about themselves or others. The open-ended survey responses provided some insight into the conditions under which participants would worry (or not) about self-isolation, such as being responsible for the care of others, the impact on mental health associated with isolation and assumptions around continued pay (Additional file 3):

“I’m a carer for vulnerable adults and would be worried about them receiving care that I could not give if I was isolating.” (survey)

Similar to the survey responses, interview participants expressed concerns regarding self-isolation such as household members becoming sick, household members potentially not being paid because they too would be affected, challenges for those with mental health difficulties or unsafe/unsuitable home environments, and worries and concerns about caring responsibilities (Additional file 3). Suggestions for how to support other household members and reduce the negative impacts of self-isolation included: granting leave that is not counted as sick leave or annual leave; offering payments if other household members would lose income; offering testing for other household members; and being flexible around specific individual circumstances.

“… [provide] more generous payments or financial support to colleagues that potentially would be quite affected by that.” (interview)

The consultation and available literature highlight the importance of adequate and tailored support: not just in terms of providing financial support for the staff member, but also thinking about the other members of the individual’s household.

### Choices about programme participation

#### Main recommendation: Carefully think about how far staff participation in testing should be mandatory or voluntary. Consider ethical issues, relevant laws, and the special nature of the employee/employer relationship

An important consideration in the public health ethics literature is the extent to which the interests of the wider public might potentially compete with the interests of individuals and impact on individuals’ autonomy [32, 55, 59]. While the tension between respecting autonomy and protecting the health of the population should not be oversimplified [114, 115], it is of significance in the context of those public health measures that are put to use to control infectious disease such as COVID-19 (e.g. case isolation, household quarantine, contact tracing) [116]. The Nuffield Council of Bioethics “Intervention Ladder” [117] provides a practical framework to highlight that more restrictive and choice-limiting measures need greater justification. Specific to workplaces is that mandatory vs. voluntary programme models must be considered in the context of existing employee-employer relationships, which typically involves some degree of power imbalance between employers and employees [118].

**Fig. 3:**
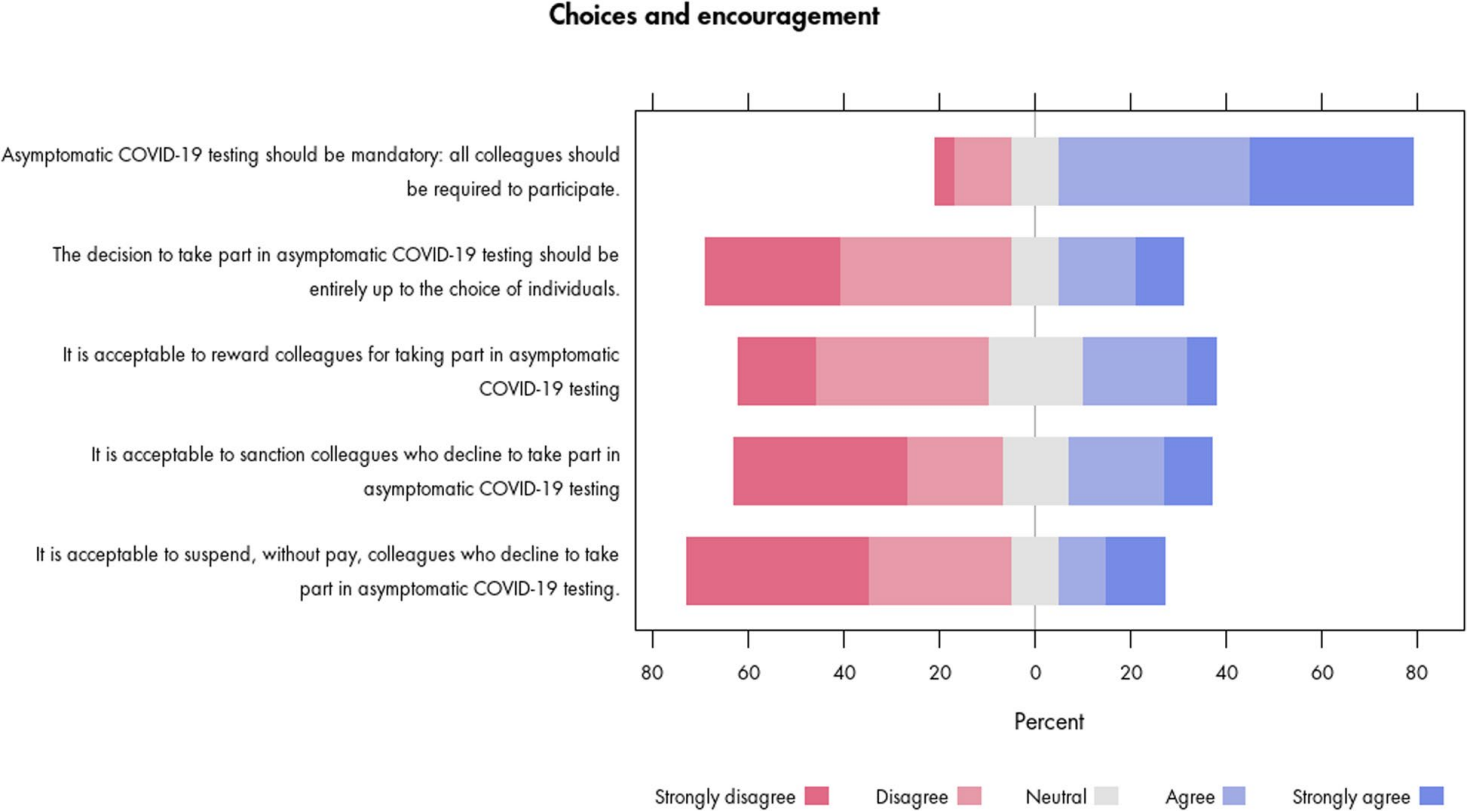
Survey participants’ views on choices, encouragement and coercion towards testing

The complexities of negotiating ethical issues around choice about participation in a testing programme were also highlighted by our consultation. For instance, the majority (74%) of survey participants agreed that asymptomatic workplace COVID-19 testing should be mandatory; similarly, 64% disagreed that it should be entirely up to the choice of individuals (Fig. 3). At the same time, around a third (30%) agreed it would be acceptable to sanction colleagues not taking part, and around a fifth (22%) agreed that it would be acceptable to suspend colleagues without pay. Some interview participants for example suggested that mandating testing might be justified to mitigate insufficient participation, but individual choice was nonetheless recognised as a key consideration.

“Covid’s a collective problem […] I have absolutely no problem with them being mandatory, and if anything, I think it’s good, because you know the expectation is the same across the board.” (interview)

“I’m a bit torn about this issue, because I do think it’s not like, morally, is it right to make someone be involved with an exercise that they don’t want to be.” (interview)

There were also divergent views (see Additional file 3) on those actions that would not make testing mandatory, but might nonetheless create a sense of compulsion (e.g. making people feel bad about not taking part, restricting their duties, temporarily stopping people’s pay by having them go on unpaid leave if they decline to take part). Some suggested that “effectively compulsory” interventions were particularly problematic because they implied choice but then penalised the ‘incorrect’ one, though participants also acknowledged it might be useful as a means to an end in encouraging people to take part in testing.

Our consultation – and public health models such as the Nuffield Intervention Ladder [117] – suggest that mandating participation could be considered in specific circumstances. Such a policy would need to be proportionate to the level of risk and benefit, achieve goals not achievable in a less intrusive way [54, 108], and consider the distinctive nature of workplaces including employer/employee/client relationships [32, 102].

### Benefits, harms and their distribution, including opportunity costs

#### Main recommendation: Assess possible benefits and risks, harms, costs, and things that you can’t do because you are doing this programme. Think about equity and whether some staff groups might bear more burdens than others

The legitimacy of a public health intervention relies on a reasonable balance between benefits and burdens or individual/societal costs [56]. An intervention associated with larger burdens requires higher levels of effectiveness to be considered legitimate [54, 59]. For example, as emphasised by a SAGE statement, asymptomatic testing programmes need to provide benefits beyond symptomatic testing to be considered worthwhile or justifiable [17]. The benefits and drawbacks of testing programmes [79] need to be considered explicitly, as do the obligations to ensure that legal requirements relating to equalities are upheld [51, 52], and that certain groups do not disproportionately suffer the negative impacts of such public health interventions [32, 51].

The vast majority of survey participants (90%) viewed a workplace asymptomatic testing programme as likely to be helpful or extremely helpful. Similarly, only 4% of participants viewed testing as unacceptable even if it would be highly effective in reducing virus transmission. For the vast majority (96%), workplace testing was deemed acceptable when it would have a small effect (32%) or be at least moderately (38%) or highly (26%) effective for reducing virus transmission. In open-ended survey responses and interviews, effectiveness, costs and resource use were mentioned as criteria to justify a workplace asymptomatic testing programme (Additional file 3).

“I don’t see any advantage really of one or two people being picked up, to me that doesn’t really … it’s pointless.” (interview)

The majority of survey participants expressed concern about negative impacts on colleagues who are worried about money or losing wages (58%) or those on short-term or temporary contracts (55%). The open-ended survey data and interviews offered further insights into these concerns (Additional file 3), including their views on potential impacts on those on lower wages or with lesser job security.

“… maybe people who are not permanent staff […] For those individuals to know how they’re going to be supported and whether or not they’re going to lose their jobs; if someone else is going to replace them who doesn’t have an issue with mandatory testing.” (interview)

Our consultation data and wider analysis suggest that organisations need to assess any possible benefits, costs and drawbacks of a testing programme, and to ensure that equity and fairness are considered carefully in any such assessment [79].

### Privacy, confidentiality and data protection

#### Main recommendation: Ensure the programme meets data protection and confidentiality requirements. Be clear who will be informed about test results and why

The measures put in place to control COVID-19 (e.g. contract tracing) may require the collection, sharing, and retention of information, which raises questions of privacy and confidentiality [32, 59]. Workplaces must process the data lawfully, securely, transparently and fairly, in accordance with legal requirements governing data protection [30, 31]. Further, organisations that run testing programmes can have obligations for notifying public health agencies about positive results [4, 5, 31, 74].

In our consultation, survey participants were very supportive of information about positive cases being shared as long as the individual who had tested positive was not identified: 96% indicated that everyone who has been in close contact with that individual should be told. In addition, there was support for use of anonymised data for scientific research: 90% of participants strongly supported this.

Confidentiality and privacy were highlighted as important by interview participants, with strong support for only a restricted number of individuals being informed of an individual’s positive result on a ‘need to know’ basis. Participants were in favour of only disclosing the name of a positive-testing employee with those who directly need the information (e.g. line manager, human resources team), but emphasised the importance of consent where possible and appropriate.

“So if that individual has had close contact with other people, then their manager should be aware of what’s happened, but the immediate question should be asked, we need to tell other people in the team because they may need to isolate, are you happy with this?” (interview)

At the same time, the risk of deductive disclosure was recognised and was a source of some concern; it was acknowledged that it would often be easy to guess who had tested positive for COVID-19 (Additional file 3).

The literature and our consultation suggest the need for a proportional response: private health information (including the name of someone who tested positive) should only be shared if there is no other way to protect other colleagues, clients and public health [32, 56].

### Communication

#### Main recommendation: Make clear communication with staff a priority, and put feedback and response mechanisms in place

Effective, transparent and open communication is regarded as key for successful public health interventions [56, 108, 119]. Despite emphasis of SAGE that COVID-19 testing programmes require communication built on trust, shared goals and perceived fairness [17], concerns have been expressed that this need has not always been met [14].

All survey participants (100%) agreed that high quality, clear and honest communication was important to building trust in a workplace asymptomatic testing programme. In open-ended survey responses and interviews, participants referred to a variety of additional information that should be communicated to participants, such as how long the programme would be in operation, transparency about results, and how contact tracing would work (Additional file 3). Clear information about how to raise concerns or make enquiries was seen as important for building trust in the testing programme.

High quality communication was seen by participants as particularly important to trustworthiness and legitimacy – for example through clear explanation of the purpose of the testing and providing insights into the reasoning behind the decision to start a testing programme. Interview participants expressed how trust can be undermined if employees are not sufficiently consulted in the design and decisions about the programme.

“I think just transparency in everything, […] because I think I would not trust it if I felt like my views were not reflected at all and if it was just a top-down decision and you’re like it doesn’t matter what you think because this is what we’ve decided.” (interview)

As our consultation and public health literature shows, high-quality communication and engagement related to COVID-19 workplace asymptomatic testing programmes is needed to build trust, address employees’ concerns and keep relevant stakeholders informed.

## Discussion

The COVID-19 pandemic has highlighted the need for ethical guidance for public health interventions, including those affecting employees [15, 43, 44]. This is particularly true for workplace testing programmes, which raise complex ethical issues. Our mixed-method consultation helped to articulate the values that may inform employees’ orientations towards testing programmes. It also helped to reveal some of the tensions and dilemmas that may need to be addressed in practice. Though there was strong staff support for the idea of an asymptomatic COVID-19 testing programme, it was not uncomplicated. Participants expressed concerns about the effects of the programme on the nature of the employer-employee relationship, the goals and scope of the programme, whether the programme would be mandatory, how support for isolation would be organised (in particular for those in less financially secure positions), and regarding privacy, data security and communication. The findings of the consultation were important in informing the ethical framework we devised to guide decision-making for COVID-19 asymptomatic testing programmes in the workplace (Table 1).

A prominent feature of our framework is its advocacy for a whole-system approach to workplace testing programmes [6, 76, 81, 120]. If organisations choose to operate testing programmes for their staff, they should recognise that they may have ethical obligations to support isolation financially and in other ways [29, 32, 113]. They should also ensure they have reliable systems for prompt notification of public health bodies of positive cases if required [4, 5, 30, 47, 74], and address their legal and ethical responsibilities for handling of data [30, 121].

A key ethical consideration – identified by participants in this consultation and in the wider public health ethics literature [59, 61, 117, 122] – is whether participation in testing programmes in workplaces should be mandatory or voluntary. One relevant consideration is the potential of testing to protect others, such as colleagues and clients of the organisation [32]. Mandatory testing for drugs and alcohol, for example, is sometimes justified on the basis that employers have legal responsibilities for health and safety risks (particularly those affecting the public) [37, 38, 123]. In the case of asymptomatic COVID-19 testing, the extent to which programmes are effective in protecting others is currently not fully clear, depending on some combination of current transmission rates, properties of the test used, and participation rates [77, 80, 82–86]. These uncertainties complicate arguments in favour of mandates.

Further uncertainty arises because, reflecting the structural dimensions of different models, a voluntary approach might resolve some issues – for example those related to perceived coercion – but potentially create others, such as unfairness. For example, some occupational groups may be much more able to opt out of testing than others: those in more elite positions or more secure employment may be much better placed to choose, for example because they are better able to choose to work from home. A programme that lacks full participation might also distribute risks unfairly. It is possible that having a testing programme in place might be particularly protective of lower-status individuals or those with direct client-facing roles. Having a programme where some employees could opt out might increase the risks to these individuals.

Organisations operating mandatory schemes should be aware of their responsibilities to ensure fairness and a climate of trust. Employee-employer relationships typically involve some degree of power imbalance, such that even formally non-mandated programmes can be experienced as coercive. This may arise because officially-communicated expectations, use of overt positive incentives (e.g. small rewards) or negative incentives (e.g. penalties). More subtle influences might arise through more covert pressures, such as peer or manager expectations [124]. Creating opportunities to understand the programme as a collective good rather than one imposed by fiat on a reluctant workforce [125] may therefore be an especially important task. Studies of other interventions have also suggested that a focus on the quality of the work environment can help to reduce concerns about autonomy and privacy [126, 127].

A linked issue is accountability for the reasonableness of the goals and scope of the programme [55, 59, 60]. Some of the concerns raised by participants related to whether the goals and operation of the testing programme were reasonable. Organisations should show their good motives and intent and demonstrate honesty, integrity, and commitment to learning. They should reflect regularly on the goals of workplace testing programmes, review whether the purposes being served by the programme are still valid and relevant to need, and ensure that goal drift has not occurred. Also clear is that any alterations to goals should be subject to consultation and communicated clearly [29], and the programme should not be subverted to serve other goals, such as using data from the programme as a means of surveillance of productivity or workplace attendance [30].

One way to approach the challenges is to commit to trustworthiness. Having trustworthy institutions will enable much to go right, enabling employees to have faith that efforts in relation to the programme are well-motivated and have their interests at heart. Thus, for example, more participants in our project saw keeping people safe as a legitimate goal for the programme than ensuring business continuity. The need for trustworthiness is one reason organisations should be clear and open in the goals of the programme and how far they can be achieved. Organisations should seek to ensure that programme goals can be recognised as legitimate, even if not everyone agrees with them. One key way to do this is through participatory governance. Including employees in the design and implementation may help to reduce possible barriers in participation, for example by ensuring a programme is meaningful to all employees [34, 128], and that preferences of employees and employers for programme components are accounted for [129]. This aligns with another key insight from the consultation: a trustworthy testing programme requires clear, transparent and accessible communication from leaders over all aspects of testing.

### Strengths and limitations

Our approach, using consultation findings, literature review, available international and national guidance, and expertise from a multidisciplinary team was a strength of this study. It offered multiple perspectives on an important problem and informed the generation of a comprehensive framework, demonstrating the strength of using participatory approaches with involvement from different stakeholders to engage with a complex (ethical) issue [54–57]. It was also a valuable approach in a parallel project focusing on asymptomatic COVID-19 testing for students in higher education institutions [23], which similarly combined stakeholder consultation with a process of reasoned and deliberative justification [72].

The study’s consultative, participatory approach is likely to help in securing legitimacy for its findings [72], but it does have limitations. Use of convenience and purposive sampling resulted in the recruitment of a diverse group of participants, but it is possible that views gathered were limited by only including staff sufficiently interested to express views. The work was done in one organisation, and we have not established the external validity of our findings; future work could focus on different settings, such as those that include people in less secure job positions – for example, those in casual employment, zero-hours contracts, or sub-contractors. The consultation was conducted at one moment during the fast-moving pandemic crisis, and views may change over time. When science and policy are moving this quickly, the principles and practices of such programmes need to be kept under review.

## Conclusions

Workplace testing programmes for asymptomatic COVID-19 infection are not free of ethical challenges and dilemmas. This study reports views of a consultation in a case study organisation that helped to inform practical and actionable recommendations for how a COVID-19 workplace asymptomatic testing programme can be set up in an ethical way (Table 1). Grounded in wider ethical thinking, public health literature and guidance, and multidisciplinary professional expertise, the framework is intended to support employers in structuring their thinking about COVID-19 testing programmes in key areas ranging from design and operation of the programme through to choices about participation. The broad principles and recommendations of the resulting framework may be applicable to various workplaces, but this requires further validation.

## Supplementary Information

**Additional file 1.** Questionnaire used in the case study consultation.

**Additional file 2.** Interview guide used in the case study consultation.

**Additional file 3.** Selection of supporting data from the survey and interviews that informed the framework.

## Data Availability

Owing to the conditions of the ethical approval for the project, the raw data (transcripts and survey responses) are not available for deposit. This is due to the sensitive nature of the responses, including their possible political nature, and concerns that it would be difficult to completely de-identify participants (who often gave extensive and specific details about their organisation and own circumstances in answering questions). Any requests for access to or use of the data should be made to director@thisinstitute.cam.ac.uk. Access to fully anonymised data for suitable purposes may be granted to bona fide researchers under a data sharing agreement, subject to approval from relevant ethics committee/s.
