# Supplementary material for: Guiding organisational decision-making about COVID-19 asymptomatic testing in workplaces: mixed-method study to inform an ethical framework
Source: BMC Public Health. 2022 Sep 15;22:1747. doi: 10.1186/s12889-022-13993-1 (PMC9476340; doi:10.1186/s12889-022-13993-1)
Supplement: Supplementary file 1 — Additional file 1. Questionnaire used in the case study consultation. [file 12889_2022_13993_MOESM1_ESM.pdf]

Choice and consent
I would like to participate in the following way:

- Complete a survey
- Schedule a live interview

To participate in this project you will need to read and agree to all of the following statements:

	Yes	No
I confirm that I am aged 18 or over.	○	○
I confirm that I have read and understand the information about this project provided on the study webpage	○	○
I have had the opportunity to email the project team at enquiries@thisinstitute.cam.ac.uk to ask any questions about the project.	○	○
I understand that some of the information I provide during this consultation might be considered as political opinion or other data that would be classed as Special Category data under Article 9 of the General Data Protection Regulation (GDPR). I consent to the use of this information for the purposes of this project.	○	○
I understand that my participation is voluntary and that I am free to withdraw at any time without giving any reason, without my legal rights being affected.	○	○
I agree that anonymised data from the consultation may be used in reports and publications arising from the project.	○	○
I understand that anonymised data may be accessed by the research team, the research sponsor (the University of Cambridge), or by regulatory authorities for the purpose of monitoring and audit only.	○	○
I understand that anonymised data from this consultation may be used to compare the different online approaches to help future projects.	○	○
I agree to my interview being digitally recorded.	○	○
I agree to take part in the project.	○	○

Introduction
We'd like to ask you some questions about workplace programmes designed to test for asymptomatic COVID-19. These programmes are used to test for possible infection in people **who do not have symptoms**, and the tests may happen regularly and may be mandatory. When you answer these questions, please think about the context of your own workplace and organisation.

1.1 Duty of care and justification
This question is about the **responsibilities** of workplaces to introduce and manage a testing programme for asymptomatic COVID-19. Please indicate how strongly you

agree or disagree with each of the statements below:

	Strongly disagree	Disagree	Neutral	Agree	Strongly Agree
**Overall**, workplaces have a responsibility to operate a COVID-19 asymptomatic testing programme.	○	○	○	○	○
Workplaces have a responsibility to operate a COVID-19 asymptomatic testing programme **to protect the health of colleagues in the organisation.**	○	○	○	○	○
Workplaces have a responsibility to operate a COVID-19 asymptomatic testing programme **to protect the health of customers/clients of the organisation.**	○	○	○	○	○
Workplaces have a responsibility to operate a COVID-19 asymptomatic testing programme **to protect broad public health.**	○	○	○	○	○
Workplaces have a responsibility to operate a COVID-19 asymptomatic testing programme so **the business can keep going in a pandemic situation.**	○	○	○	○	○

Please offer any comments you may have on the responsibilities of workplaces to provide asymptomatic testing.

This question is about how **effective** a testing programme of asymptomatic COVID-19 needs to be in order for it to be acceptable. Please choose one of the following:

○ Even a **small effect** on virus transmission of asymptomatic workplace testing programmes in the workplace makes such programmes **acceptable**.

○ To be **acceptable**, asymptomatic workplace testing programmes should be **at least moderately effective** in reducing virus transmission.

○ To be **acceptable**, asymptomatic workplace testing programmes should be **highly effective** in reducing virus transmission.

○ Even if asymptomatic workplace testing programmes are highly effective in reducing virus transmission, **they are not acceptable**.

Please provide any comments you may have on the effectiveness of testing programmes and their acceptability.

1.2 Privacy, confidentiality and data protection

If you were tested for asymptomatic COVID-19 in your workplace, how **acceptable** would you find the following ways of **letting you know the results**?

	Completely unacceptable	Unacceptable	Neutral	Acceptable	Completely acceptable
Email to my work email address	○	○	○	○	○
Email to my personal email address	○	○	○	○	○
Text message to my work phone	○	○	○	○	○
Text message to my personal phone	○	○	○	○	○
Phone call to my work phone	○	○	○	○	○
Phone call to my personal phone	○	○	○	○	○
An online facility I can log into to get my responses	○	○	○	○	○

Are there any other ways you would prefer to be told about your test results?

Who should be told automatically when there is a positive test in the workplace, but **without being told the name** of the person who has tested positive? Please tick as many as you like

- ☐ The person's line manager
- ☐ Occupational health services (if available)
- ☐ Everyone the person has been in close contact with in the workplace
- ☐ Everyone the person works with, whether or not there has been close contact
- ☐ Other (please say who):
- ☐ No one should be told

If someone in the workplace tests positive through the asymptomatic testing programme, who do you think should automatically be told **the name of the person**?

Please tick as many as you like.

- ☐ The person's line manager
- ☐ Occupational health services (if available)
- ☐ Everyone the person has been in close contact with in the workplace
- ☐ Everyone the person works with, whether or not there has been close contact
- ☐ Other, please say who: __________
- ☐ No one should be told

Data from the asymptomatic testing programme might have value for other purposes, such as scientific research about COVID-19, making estimates of workplace infection rates, and monitoring and evaluation of the programme. Any data used for such purposes would have all identifying details removed, to ensure anonymity of those tested in the programme. Please indicate your **level of support for using test data** for the following purposes:

	Strongly oppose	Somewhat oppose	Neither oppose nor support	Somewhat support	Strongly support
Using the data for **scientific research about COVID-19**	○	○	○	○	○
Using the data for **making estimates of workplace infection rates**	○	○	○	○	○
Using the data for **monitoring and evaluation** of the programme	○	○	○	○	○

Would you support keeping test data (without your identifying details) for 20 years for these purposes?

- ○ Yes, 20 years is ok
- ○ No, 20 years is too long
- ○ No, 20 years is too short
- ○ No, my test should not be kept at all

Please add any comments that will help us understand your views:

1.3 Worries and concerns about the test

This question is about potential **worries and concerns** related to the testing programme. Please indicate how strongly you agree or disagree with the following statements:

	Strongly disagree	Disagree	Neutral	Agree	Strongly agree
**Testing properties**					
I am concerned about the possible discomfort of having the test.	○	○	○	○	○
I am concerned that the test could give a false positive result (it might say I have COVID-19 when really I do not).	○	○	○	○	○
I am concerned that the test could give a false negative result (it might say I do not have COVID-19 when really I do).	○	○	○	○	○
**Taking part**					
I am concerned that not everyone will take part, which could decrease the value of the programme for reducing infection.	○	○	○	○	○
I am concerned that I might feel forced to take part even when I don't want to.	○	○	○	○	○
**Information**					
I am concerned about who would know whether or not I take part.	○	○	○	○	○
I am concerned about who would know about my test results.	○	○	○	○	○
I am concerned that information about me collected as part of the programme could be used for a purpose I don't agree with.	○	○	○	○	○
**Impact**					
I am concerned that the programme could have particularly negative impacts on colleagues from ethnic minority groups.	○	○	○	○	○
I am concerned that the programme could have particularly negative impacts on colleagues who are worried about money or losing wages.	○	○	○	○	○

	Strongly disagree	Disagree	Neutral	Agree	Strongly agree
I am concerned that the programme could have particularly negative impacts on colleagues who are on short-term or temporary contracts.	○	○	○	○	○

If you test positive you will not be allowed to come to work for 10 days (if symptom-free). You would be asked to self-isolate, along with other members of your household if you live with others. Please tell us how strongly you disagree or agree with the following statements.

	Strongly Disagree	Disagree	Neutral	Agree	Strongly agree
If I had to isolate, I would worry about possible loss of income for myself.	○	○	○	○	○
If I had to isolate, I would worry about struggling with practical issues (e.g. groceries, childcare).	○	○	○	○	○
If my household has to isolate because I have a positive test, I would worry about the impact on my household (e.g. children missing school, missing family events, other members of the household missing work).	○	○	○	○	○
If I had to isolate, I would worry about possible impact on my mental health.	○	○	○	○	○

Please tell us about **any other worries or concerns you personally have** about the asymptomatic COVID-19 testing programme for your organisation.

1.4 Prioritising testing

For each statement below, please indicate how strongly you agree or disagree about **how resources for testing should be prioritised.**

	Strongly disagree	Disagree	Neutral	Agree	Strongly agree
In a situation where capacity for testing is limited, asymptomatic testing should be prioritised for people who are at higher risk (for example because of age).	○	○	○	○	○

	Strongly disagree	Disagree	Neutral	Agree	Strongly agree
In a situation where capacity for testing is limited, asymptomatic testing should be prioritised for people who are in higher risk roles (for example because they are in contact with members of the public).	○	○	○	○	○
Asymptomatic testing for people in specific workplaces should only be conducted if there is enough testing capacity available to people in the general public who are having symptoms.	○	○	○	○	○

1.5 Information about the programme
Please tell us how **important** it is to you to be given **information** about the each of the following aspects of the programme.

	Very unimportant	Unimportant	Neutral	Important	Very Important
What a test involves, including details of any discomfort I might expect	○	○	○	○	○
Accuracy of the test	○	○	○	○	○
How long it takes to get the test results	○	○	○	○	○
How I will be told about the test results	○	○	○	○	○
Who else will be told about the test results besides me	○	○	○	○	○
What support is available if I have to isolate (for example, paid sick-leave)	○	○	○	○	○
If anonymised results will be used in scientific research, monitoring or evaluation	○	○	○	○	○
Whether updates about how many people test positive in the workplace (e.g. 1 in 100; 1 in 1000) will be provided regularly	○	○	○	○	○

What other information, if any, would you like about the programme?

1.6 Choices regarding testing
For each statement below, please indicate how strongly you agree or disagree about **requirements to participate** in the COVID-19 asymptomatic testing programme.

	Strongly disagree	Disagree	Neutral	Agree	Strongly agree
Asymptomatic COVID-19 testing should be mandatory: **all colleagues should be required to participate.**	○	○	○	○	○
The decision to take part in asymptomatic COVID-19 testing should be **entirely up to the choice of individuals.**	○	○	○	○	○
**It is acceptable to reward colleagues** for taking part in asymptomatic COVID-19 testing (for example by giving them catering vouchers).	○	○	○	○	○
**It is acceptable to sanction colleagues** who decline to take part in asymptomatic COVID-19 testing (for example being excluded from rewards and benefits schemes).	○	○	○	○	○
**It is acceptable to suspend, without pay, colleagues** who decline to take part in asymptomatic COVID-19 testing.	○	○	○	○	○

1.7 Trustworthiness and legitimacy

Please indicate how important the following would be for **having trust in the programme.**

	Very unimportant	Unimportant	Neutral	Important	Very importan
High quality, clear and honest information.	○	○	○	○	○
Reliable, fast communication of test results.	○	○	○	○	○
Confidentiality about whether I have been tested or not.	○	○	○	○	○
Confidentiality of my test results.	○	○	○	○	○
Reassurance that testing positive will have no impact on my job or career.	○	○	○	○	○
Practical help with isolation if I test positive.	○	○	○	○	○
Mental health help with isolation if I test positive.	○	○	○	○	○
A way of anonymously raising any concerns I have about the programme.	○	○	○	○	○
Allowing anonymised results of the testing programme to be used in scientific research.	○	○	○	○	○

Is there anything else that would help you with trust in the programme?

Is there anything that would damage your trust in the programme?

1.8 Benefits and harms

Please rate your view of **how helpful** the programme is likely to be, **overall**:

Extremely unhelpful	Unhelpful	Neutral	Helpful	Extremely helpful
○	○	○	○	○

Please list below **any possible upsides** of having an asymptomatic COVID-19 testing programme for your organisation.

Please list below **any possible downsides** of having an asymptomatic COVID-19 testing programme for your organisation.

Concerns or Ethical issues

Are there any other **concerns or ethical issues** about the testing programme that you would like to raise?

Demographics intro

We would like to learn a little bit more about your role within the organisation and your background, so that we can better understand the views of different people across the organisation. We would appreciate if you could answer the following last few questions. After that, the survey is done.

1.9 Demographics and staff role

Please select one of the following options that most closely matches your **staff role**.

- Higher managerial, administrative or professional occupation
- Intermediate managerial, administrative or professional occupation
- Supervisory or clerical and junior managerial, administrative or professional occupation
- Skilled manual worker
- Semi-skilled and unskilled manual worker
- Casual and lower grade worker
- Student, volunteer, intern

Please select one of the following options that best describes your type of **work contract/arrangement**:

- Permanent contract with the organisation in which you work
- Fixed-term contract with the organisation in which you work
- Contract with a different employing organisation, such as an agency
- Self-employed (e.g. freelancer, consultant, contractor)
- Unpaid (e.g. student, volunteer, intern)

How many **hours per week** do you work for or at the organisation?

- Full-time
-

Part-time (please provide the number of hours per week)

○ No fixed number of hours (including zero-hour contracts)

When you are in your workplace (for example during the pandemic), are you **regularly in close contact** with members of the public, clients, customers or other people external to the organisation?

○ Regularly in close contact with more than 20 people over the course of a day at work
○ Regularly in close contact with around 10-20 people over the course of a day at work
○ Regularly in close contact with 1-9 people over the course of the day at work
○ Rarely or never in close contact with other people over the course of the day at work

Do you **care for others** outside your work, such as children, a partner, relative or friend?

○ Yes
○ No
○ Prefer not to say

Current **age** in years:

○ Current age in years

○ Prefer not to say

What **gender** do you identify as?

○ Woman (including transgender woman)
○ Man (including transgender man)
○ Prefer not to say
○ Prefer to describe myself as (e.g. non-binary, gender-fluid, agender):

What **ethnic group** do you identify as?

White

- English / Welsh / Scottish / Northern Irish / British
- Irish
- Gypsy or Irish Traveller
- Any other White background

Mixed / Multiple ethnic groups

- White and Black Caribbean
- White and Black African
- White and Asian
- Any other Mixed / multiple ethnic background

Asian / Asian British

- Indian
- Pakistani
- Bangladeshi
- Chinese
- Any other Asian background

Black / African / Caribbean / Black British

- African
- Caribbean
- Any other Black / African / Caribbean background

Other ethnic group

- Arab
- Any other ethnic group

Prefer not to say

- Prefer not to say

Do you have a **longstanding (more than 6 months) illness, disability or impairment** which causes substantial difficulty with day-to-day activities?

- Yes
- No
- Prefer not to say

Do you have an illness, disability, or impairment which is likely to put you at **increased risk from COVID-19**?

- Yes
- No
- Not sure
- Prefer not to say

Demographic Info Interlude for Interview Pathway

Thank you for choosing to participate in an online interview. We would like to learn a little more about you, to make sure we can interview different people from across the

organisation. **Please respond to the questions below.** Our research team will contact you within two working days about an interview.

Powered by Qualtrics
