# Supplementary material for: Guiding organisational decision-making about COVID-19 asymptomatic testing in workplaces: mixed-method study to inform an ethical framework
Source: BMC Public Health. 2022 Sep 15;22:1747. doi: 10.1186/s12889-022-13993-1 (PMC9476340; doi:10.1186/s12889-022-13993-1)
Supplement: Supplementary file 2 — Additional file 2. Interview guide used in the case study consultation. [file 12889_2022_13993_MOESM2_ESM.pdf]

THIS.Institute The Healthcare Improvement Studies Institute

Made possible by The Health Foundation

Workplace testing framework project

Interview guide

Version date: 2020-11-10

I'd like to talk to you today about asymptomatic workplace testing programmes, which test colleagues for COVID-19 when they are NOT showing symptoms of the disease and may be mandatory. The interview will take about 30 to 45 minutes, but we can stop at any time before that if you wish to do so. When you answer these questions, please think about the context of your own workplace and organisation.

Introduction

(1) Could you tell me a little bit about yourself, for example about your current role in the organisation you're working at?
- What's changed about your role since the pandemic, if anything?
- What has working more at home been like for you?
- How have you felt about these changes?

(2) Have you heard about the introduction of the mandatory asymptomatic testing programme for COVID-19 at your organisation? What was your first reaction when you heard about it?

Goals of the asymptomatic testing programme

(3) Thinking about the goals of the testing programme, what do you think it is trying to achieve?
- Would you see these are reasonable goals for the programme?
- Can you see any reasons why it might not be possible to achieve those goals?

Support

(4) Let's suppose you test positive, you will not be allowed to come to work. You would also be asked to self-isolate, and perhaps also other members of your household, if you live with others. Can you tell us how you would feel about that?
- What worries or concerns would you have for yourself?
- And for others in your household?

(5) What kind of support from the organisation would be helpful if you have to isolate after a positive test?
- For example, paid leave?

UNIVERSITY OF CAMBRIDGE

Test properties

(6) Unfortunately it's scientifically not possible to be 100% sure that a positive test means that someone has COVID-19, or that a negative test means that someone is not infected. What are your views on having to self-isolate, and perhaps also members of your household, but not knowing 100% sure if the test was correct?

Duty of care and justification

(7) Would you say there is a responsibility for organisations to introduce asymptomatic testing programmes if they are able to resource it? Why is that?
(8) No testing programme is going to be completely successful in reducing virus transmission. Can you share your views on how effective the programme would need to be to justify having it?

Mandatory testing

(9) The programme will be mandatory for all colleagues who are working in organisation's buildings rather than at home. How do you feel about that?
- What would you say to people who say that the decision about taking part in asymptomatic testing should be entirely up to individuals?
- What about actions that don't make testing mandatory, but that might still feel coercive – like making people feel bad about not taking part or restricting the duties they do?
- What about temporarily stopping people's pay by having them go on unpaid leave if they decline to take part in the testing programme?

Access to testing

(10) It could be that sometimes there will be limits on testing capacity in the country as a whole, so not enough tests are available on time for everyone who wants one. How would you feel, for example, if it's hard for members of the public to get tests if they DO have symptoms, but your organisation still offers tests to all colleagues even though they don't have symptoms?

Inclusion and diversity

(11) Do you think there are colleagues or groups of colleagues who might be particularly negatively impacted by the asymptomatic testing programme?
- You could for example think about people with less job security, those at higher risk if they get COVID, people with certain ethnic backgrounds, or those who don't have high incomes.
- Why is that?
- What should be done to help them?

(12) Do you feel personally at any disadvantage from the testing programme because of your ethnicity, age, or sex, or anything else?
- Could you say a bit more about why you feel that way?

Communication

(13) What kind of information do you think should be provided to colleagues about the programme?
- How should it be communicated?
- What is the best way of making sure that the information about the programme gets to everybody in the organisation and is understood by everybody?

(14) What would be your preferred way of getting test results – for example text message, phone call, email, online service?
- Does it matter who the results come from? Why is that?

(15) What should colleagues be told about the results in the organisation overall – for example how many positive tests have been found each week?
- Is there anything else that should be told about the results in the organisation overall?

Privacy, confidentiality and data protection

(16) What information should be available about the results of unnamed individuals – so you would know that someone had tested positive in a particular area, but not who it is?

(17) If a named colleague tests positive, who should be told automatically?

(18) Who should be allowed to know about a named colleague's result if they ask?

(19) How would you feel about data from the testing programme being used for purposes like scientific research, monitoring of infection rates etc, if the identifying details were removed?

Worries and concerns, upsides and downsides

(20) Thinking about everything we talked about, what advantages can you see of the programme? What disadvantages can you see?

(21) Thinking about everything we talked about, what would worry or concern you most about the testing programme?
- What could be done to help with these worries and concerns?

(22) What would be the best way to enable colleagues to raise concerns about the testing programme if they have them?

(23) Thinking about everything we talked about, what are the things that would help you have trust in the programme? What kinds of things would damage trust?

Other concerns

(24) Are there any other concerns or ethical issues about the testing programme that you would like to raise?
