# Supplementary material for: Guiding organisational decision-making about COVID-19 asymptomatic testing in workplaces: mixed-method study to inform an ethical framework
Source: BMC Public Health. 2022 Sep 15;22:1747. doi: 10.1186/s12889-022-13993-1 (PMC9476340; doi:10.1186/s12889-022-13993-1)
Supplement: Supplementary file 3 — Additional file 3. Selection of supporting data from the survey and interviews that informed the framework. [file 12889_2022_13993_MOESM3_ESM.pdf]

Additional file 3. Selection of supporting data from the survey and interviews that informed the framework

Ethical consideration	Open-ended survey responses	Interviews
**1. Design and operation of the programme**	Nature, size and resources of the organisation matter when considering a responsibility:<br><br>*"... it [testing] can easily be adopted by large organisations who have increased buying power, but it puts a significant financial strain on small to medium enterprises who are already struggling."*<br><br>*"... a clear and logical rule would be, if you want employees to come to the workplace, then the employer should provide regular asymptomatic testing."*<br><br>Importance of a whole-system approach around testing:<br><br>*"It's not the testing that's key, but having an appropriate follow-up strategy in the event of a positive case - enhanced cleaning, review of procedures, requesting close contact colleagues to isolate."*<br><br>*"... testing can't be the only measure put in place to protect colleagues in the workplace; social distancing and mask wearing would be sensible too."*	Nature, size and resources of the organisation matter when considering a responsibility:<br><br>*"... if [the organisation] is resource-able then [the testing programme] should happen but, you know, I'm not necessarily going to expect my corner-shop to be having the same process."*<br><br>Some concerns about testing being the right choice for their organisation; testing seen as reasonable for those who wanted or needed to work in the organisation's buildings, or to ensure business continuity:<br><br>*"I can see other job roles where it's important but end of the day, our building was open for a small number of people who were struggling a bit maybe and also a [building name], which is not a necessity."*<br><br>*"... you also introduce more work to the organisation in terms of track and trace, I would say, even though it's not track and trace."*<br><br>*"... if I were to be working in the building I would say that was something that was a good idea."*
**2. Goals of the testing programme**	Views towards organisational responsibilities and goals of a testing programme:<br><br>*"In general, I think workplaces have a higher responsibility to employees than to customers/clients. Employees*	Testing could help to reduce risks for colleagues, clients and the wider public, perhaps facilitate business continuity, and provide reassurance to those coming into the buildings:<br><br>*"I think I expect them [my organisation] to put our health*

	often don't have a choice about whether they come in [...]; customers and clients are often able to choose to minimise their risk by staying home."<br><br>*"Ensuring business continuity is the responsibility, which may involve COVID-19 testing, or it may not, depending on the nature of the business."*<br><br>Other benefits identified by the participants that could be regarded as secondary goals of the testing programme:<br><br>*"Aside from the main one that we would be able to prevent transmission across the organisation, it would probably reduce anxiety among colleagues and mean that we aren't as worried about being in close proximity during the day."*<br><br>*"As a person in a clinically vulnerable category, it helps to make the difference in feeling safe enough to return to work onsite."*<br><br>*"Reduced stress levels and improved mental health resulting from people knowing they don't have the virus."*<br><br>*"Greater belief/trust that my organisation cares about my health over my productivity."*	*and safety as a paramount priority, and so it really demonstrated to me that that's what they were doing [...] really important to give staff that reassurance, particularly those who are in more at risk age groups and with long term health conditions."*<br><br>*"... look after the members of the public who are coming in to the [building name] and I guess look after, sort of, the society."*<br><br>*"I thought it was good to help people who might feel a bit more uncomfortable about coming back, that it might help them a little bit."*<br><br>Need for clarity about goals and to avoid goal confusion or goal drift:<br><br>*"They [the organisation] can fill the building more if they say they're providing testing to everyone, so it might allow a kind of semi-return to normality."*
**3. Properties of the test(s) selected for the programme**	Experiences and concerns about the test:<br><br>*"No point testing if it takes a long time to get results - the point of testing is to have a quick check and enable anyone who might be infected to leave and go straight home, thus protecting everyone else. The*	If testing not sufficiently accurate, then a second test to confirm a result:<br><br>*"... it would be really frustrating if I got a test that came back as a false positive and I isolated for two weeks, and it was a waste of time, but do you know what, the tests...perhaps the other 99 out of 100 tests that have gone to someone else have been correct.*

	whole thing falls apart otherwise."<br><br>*"The test is uncomfortable to take [...] Hard to remember to take the test - a new part of your routine [...] Stress over taking the test wrong/ forgetting."*<br><br>*"Any concerns about the test and other practicalities are outweighed by the benefits of being able to limit the spread of the virus."*<br><br>*"... if testing is done on the way into the building but an employee is then at their desk for 1-2 days before receiving a positive result, how effective is this regime relative to quick turn around tests so that an employee couldn't enter the building without a same day negative test."*	*So in the grand scheme of things, it's a really small sacrifice."*<br><br>*"... there are ways that you can really increase your confidence in those tests. So even double testing positives is a great way, and immediately, you know, you bring that false positive rate right down. So I'd have a lot more confidence."*<br><br>*"If it is like 95 per cent positive and the false positive rate is 5 per cent then I think I'm comfortable with that statistically. If it is actually 80 per cent accurate I wouldn't be very happy with it; I would probably want to have another test to check."*<br><br>Concerns expressed about correct procedures of self-swabbing, potential discomfort, invasiveness of the test, and the test's resemblances to a medical procedure:<br><br>*"I'm not sure that I did it properly myself."*<br><br>*"... it's about a swab that goes up your nose, it's about an invasive procedure."*<br><br>*"... it's a pretty intrusive test [...] so I think I would just get really fed up with it [...] they're like, shoving things down your throat. So I think I would just naturally be annoyed, if it wasn't very effective at catching it, and reducing transmission."*<br><br>*"The frequency at which it was done I think was of concern. I think every week...having to, sort of, have this test every week and...well, it's a medical procedure, having a medical procedure every week just to make sure you can come into the building was of concern."*

		Issues around the type of test:<br><br>*"I know we did have some hiccups where actually test results were taking longer [...] to reach staff, and people were worrying why was that – was there just a back log in the test lab, have I been tested positive and now they're trying to figure out what do with me?"*<br><br>*"... with some of the newer tests coming onto the kind of market, so to speak, I think there's still questions around what type of test will be used. ... the antigen test really only picks up positivity of a short duration when you've got highest viral levels. But PCR, there's some, like, six weeks later you're testing positive, even though you're not infectious."*
**4. Enabling isolation**	Conditions under which participants would worry (or not) about self-isolation:<br><br>*"As I am a young female living in a secure job with my partner I do not need to worry about issues such as childcare, passing it on to family members and job security. The biggest impact would be upon my mental health due to already suffering with depression/anxiety in the past."*<br><br>*"I am genuinely terrified about what would happen to me if I was forced to isolate and I do not feel confident that my workplace would provide the mental health support I needed if this happened."*<br><br>*"I am in a more fortunate position than others when it comes to isolation, I can work from home and do not have any children so I know the impact of isolation for me is much less than others.*	Worries related to having to self-isolate:<br><br>*"Obviously if you get a positive test and then you go home, that doesn't necessarily protect your family, so that is a tricky thing."*<br><br>*"If I'm being asked to isolate at home, what circumstances does that happen under? I mean, I have to support my little one as well so actually if it is the case that I have got COVID, then actually am I going to be passing it on to other people in my household? Are they going to be able to get a test, what does it mean for them and supporting my little one? Does that mean that I need to send my wife and my child away to my in-laws to have them isolate?"*<br><br>*"... as soon as you're self-isolating, getting ill, then you're thinking of job security as well because we know people are*

	Repeated periods of isolation would impact my mental health."<br><br>*"Impact of isolation varies significantly depending on nature of someone's work and contract, living situation, etc. The same is true for remote working. This needs to be factored into guidance in order to ensure both compliance and wellbeing."*<br><br>*"No additional worries provided that the employer continued to pay full salary and that managers were accommodating to working at home (not a problem at the moment but could be if the majority have returned to the building) and not excluded from meetings etc due to working remotely."*<br><br>*"When my workplace reopened in [date], staff on zero hours contracts were not informed about COVID related isolation/sick pay [...] Certain members of staff remain uninformed about the impact of a positive test result either for them or someone they were in close contact with."*	*losing money and losing jobs. And I think that's...you know, how long can my organisation keep up the good work of paying people and all that? [...] it's a long time to self-isolate [...] because I've got children in school and that. And I'm supporting my daughter who lives in a different house, she's in my support bubble but she's got [mental illness]."*<br><br>Suggestions put forward for how to support other household members and reduce the negative impacts of self-isolation:<br><br>*"I would definitely expect there to be, like, paid leave, or annual leave, that wasn't taken from my, like, yearly holiday time that I had. I think that feels fair to me."*<br><br>*"... if you have to self-isolate, and you're living with somebody who also has to, and they won't get compensated by their work, then maybe looking into some way of helping to compensate them for that, or giving the person a bonus [...] I think that that's something that should at least be very seriously looked at."*<br><br>*"... employers and the government would need to be providing appropriate support for people, because you don't want people to not want to get tested, because it can impact their livelihood."*<br><br>*"... offer maybe things like tests for others in the household would be quite a nice thing to be able to have, just to provide a bit of reassurance around what's happening."*

		"… from the I guess mental health wellbeing and caring point of view it would be nice if I guess you don't feel isolated and you feel well supported by the organisation … It literally could just be like a formal check in with the manager and acknowledgement that things need to be flexible."
**5. Choices regarding participation in testing programmes**	When and in what situations mandatory testing could be a viable option:<br><br>*"I think if testing is mandatory people will feel much safer about coming into the building, particularly those who are in "at risk" categories. […] I also think that making testing mandatory is much fairer to all employees as long as people don't have medical reasons why they can't take a test."*<br><br>*"Asymptomatic testing is a burden on staff and expensive and would need to be mandatory and accurate to be effective."*<br><br>*"Asymptomatic testing should be mandatory and as often as symptomatic."*<br><br>*"If a scheme is not mandatory then it will be of limited benefit. If it's mandatory then there is no issue of confidentiality about who has been tested."*<br><br>Concerns about mandatory vs testing:<br><br>*"The fact that individual choice would be removed from the equation and the employer would have a vested interest in your health."*<br><br>*"If voluntary could generate bad feeling between those that think*	On mandating the testing for those in the office:<br><br>*"The mandatory aspect of it doesn't really bother me that much because it's like...because it's protecting other people is the difficulty. […] it's other people's choices that are impacting your safety. […] if it's a seatbelt then you put on your own seatbelt and you protect yourself but, like, with testing when it's like by getting yourself tested you're actually protecting other people. So that's where I, kind of, think it's probably good that it's mandatory."*<br><br>*"Again, it's an interesting balance. I think it's...without having a vaccine that is available to everybody and having rapid testing […], I think it's the best that we've got. […] you've got responsibility to other people to be tested and, yeah, and make sure that the building and other people are safe. So, I've made my peace with that; I know a lot of people haven't. And they would say, […] can somebody else do what I'm meant to be doing inside the building, someone else who doesn't have the same concerns that I do?"*<br><br>*"For me, one of the issues is that, you know, this, like, it's an inherent concept of informed consent that's been developed*

	it's a good idea and those that done. If mandatory could anger staff that are not happy with testing."<br><br>*"If for any reason there are people who are strongly against asymptomatic testing then they might feel victimised or scapegoated in some way. I don't think this would be deliberate but the people might somehow feel this way."*<br><br>*"Different treatment of individuals across the organisation (ie mandatory for some and not others [...])."*<br><br>*"Assurance that special circumstances will be taken into account [...] we shouldn't be sanctioning someone who, for example, has a legitimate reason they can't take a test. I would hate to think we were removing pay from someone vulnerable who didn't test for justified reasons - but equally, if someone was happily spreading the virus, this should be a disciplinary issue."*<br><br>*"Not effectively taking action if people aren't taking part in a mandatory programme."*	*over, you know, hundreds of years, and that is, you know, a really integral part of research, is just, it's completely gone out the window on this."*<br><br>On requiring staff to take unpaid leave if not tested:<br><br>*"They included that additional sentence about, required to take unpaid leave if they cannot work from home [...] there'll be a small sub-group, who essentially either get tested, or lose out on income. And you know, you might as well say that you can't work here if you don't get testing, is what they're saying."*<br><br>*"I just think then you're going down the route of like...that does sound very unethical, yeah. It is a really tough one though...imagine if it's somebody that didn't want to take part because they didn't actually believe coronavirus was real [...] okay, they have a right to have that belief, but they don't really have a right to be putting everybody else at work at risk because of that belief."*<br><br>*"That would be really hard. I don't know how I would feel about implementing such a thing. But I can't see how else you have something that's mandatory if it doesn't have some sort of repercussion if someone refuses it."*<br><br>On coercion staff to take part in testing:<br><br>*"I guess in a way that's worse. I don't think somebody should be made to feel like bad. If you're going to say that it's not mandatory, it's optional, you*

		can't then make someone feel bad for taking an option that you as the employer have presented as a viable option to them."<br><br>*"I feel like you should be made to feel bad for not taking part, like you should be made to feel bad for not wearing a mask in a shop, you should be made to feel bad for having more than three families in a bubble over Christmas. Like, you know, these are basic protection measures that are actually much less strict than could be imposed. It's about protecting the country and it's about protecting much more vulnerable people and if you're not willing to do that... Like, if someone's drunk-drive I wouldn't be there, like, oh I don't want to make them feel coerced into thinking that's a bad thing. I want them to know it's a bad thing."*<br><br>*"It's, kind of like, nudging people to try and make the decision...like, it's...I'd rather policies were quite clear than, like, be ambiguous coercive...like, unwritten policy. [...] if someone isn't participating in the process [...] it might build up resentment in the workforce and it [...] it might not be institutionally but culturally those, kind of, behaviours might be adopted. [...] I can see it being a consequence potentially to a lack of maybe clearly defined policy and good communication around that."*
**6. Benefits, harms and their distribution,**	How effective a testing programme would need to be in order to be acceptable/justified:	How effective a testing programme would need to be in order to be acceptable/justified:

**including opportunity costs**	*"Workplace testing is quite an undertaking. If there was little to no effect it would be a waste of resources."*<br><br>*"We have an obligation to humanity to prevent the spread, and part of this is testing - but it needs to offer some level of effectiveness. A small effect makes this measure merely "security theatre" - something to make people feel safe without really making much difference."*<br><br>*"Personally, I feel that it would only be acceptable to test in the workplace if the tests are effective. Firstly, because it takes a large amount of resources to support a testing programme and one which is ineffective doesn't offer any value. Secondly, because as a colleague travelling to the office I would want assurance that the programme is effective at catching cases and reducing the transmission risk. There is the alternative logic, that even a partially effective programme would be good as it would still catch some cases and reduce transmission. In a workplace, where testing is not essential - people can work from home - then I think it is only acceptable if fully or highly effective. It has to be a sustainable approach in my opinion."*<br><br>*"Even a small effect in blocking the virus will accumulate as even if one extra person is infected they could spread it to two others and then on to four from there and on and on. So even catching one person with the virus may save many lives and prevent many families from suffering the pain of having their*	*"I guess it needs to be effective enough so that people can go back to their workplace, and feel comfortable, and not like, at a high risk situation."*<br><br>Potential benefits and harms of testing programmes:<br><br>*"I think keeping people safe, keeping infection rates low, being able to actually identify if someone…I know that they've made measures so if you tested positive you need to go home immediately and isolate et cetera. But then also I think it is trying to achieve operationally shifting towards getting the workplace back to normal …"*<br><br>*"If they had to have those [work]places open, it was good for the people who had to work there to have it done and to make sure that they and their families were safe and that, you know, the spreading could only be contained if you did happen to have it."*<br><br>*"I just don't think it's appropriate for workplaces to push people to come back before they're vaccinated, if there isn't an impact on their ability to do their jobs, or if there isn't a significant impact."*<br><br>Distributions of harms and burdens of testing across different groups:<br><br>*"I think, yeah, anyone with a caring responsibility, anyone who lives with somebody who doesn't have an option to work from home, and whose organisation maybe wouldn't pay for their compensation, I think that would be difficult. Anyone whose home environment wasn't safe for*

	loves ones taken away from them.”<br><br>*“To justify the cost of asymptomatic workplace testing, the programme needs to be highly effective and not replace other interventions like hand washing and mask wearing.”*<br><br>*“... anything that reduces spread is good and acceptable. This is especially true if the workplace puts colleagues at increased risk”.*<br><br>Potential benefits and harms of testing programmes:<br><br>*“Will improve the reputation of the organisation between them and employees, contractors, clients etc.”*<br><br>*“Asymptomatic testing may however encourage more people to travel to and work in an office environment. This increases the risk that they catch the virus on their journey - thereby not protecting colleagues nor members of the public.”*	*them, I think, would also be really bad, if they had, if they felt that they had to self-isolate, or they were told that they had to.”*<br><br>*“... what if their partner or flat mate is high risk then do you extend the same? Because technically you should because the partners of shielding people should be shielding as well. But I think that’s where the support should be given and there should be a real individual conversation on what’s appropriate for that person’s circumstance.”*
**7. Privacy, confidentiality and data-sharing**	With whom should anonymous results be shared:<br><br>*“There should be a clearly responsible and properly trained person who makes the decision on who needs to be contacted. It should not sit with line managers or anyone else who might not understand the process and implications of a positive test.”*<br><br>*“People who have been in close contact though I am not sure how feasible that is without some bluetooth app like the national track and trace.”*	How to communicate test results:<br><br>*“I would assume that the results are coming from whatever lab you were getting the tests analysed at. I don't think that, like, I don't think I'd be cool with having my test analysed, and then like sending it to my manager ...”*<br><br>*“There’s no point in doing it if you’re going to wait three or four days or any delay, it’s pointless.”*<br><br>How to inform staff about positive tests :

	With whom identifiable results might be shared:<br><br>*“Whoever is managing the internal track and trace process.”*<br><br>*“The (trained) designated individuals or group coordinating Covid response.”*<br><br>Concerns about confidentiality (e.g. what might damage trust in the programme):<br><br>*“Broken confidentiality.”*<br><br>*“If my name or test results were shared without my permission.”*<br><br>*“Naming and shaming people publicly.”*<br><br>*“Any perceived leak of personal data or use of data for different purposes without explicit consent could cause a breakdown of trust.”*<br><br>*“There are also confidentiality issues as a positive result would be obvious to colleagues.”*<br><br>*“If it's mandatory then there is no issue of confidentiality about who has been tested - only of the results although in fact everyone would be able to work out who had tested positive by their absence and triangulating who had been sent home as a contact.”*<br><br>Use and storage of anonymised test data (e.g. research):<br><br>*“It's a global pandemic, how could anyone refuse to allow their data to be used to help understand and stop it?”*<br><br>*“I don't see any reasonable concern that would prevent this*	*“I think their line manager should know, but it has to be with their consent. I think, again, there’s a fine line between safeguarding because it depends on what the circumstance it’s in. So if that individual has had close contact with other people, then their manager should be aware of what’s happened, but the immediate question should be asked, we need to tell other people in the team because they may need to isolate, are you happy with this? How do you want us to do it – do you want to tell them yourself; do you want to have a conversation, do we want to have a team meeting? I think all of those things need to be openly aired and discussed with that individual, but it should be done with their consent, everything needs to be done with their consent.”*<br><br>*“I guess their line ... like, whoever their line manager is [...] and then I guess anyone who’s been in contact with them maybe.”*<br><br>Sharing overall information about infection rate:<br><br>*“Yes, I think transparency is pretty good, again for people who don’t want to get tested, that transparency if there was a lot of positive tests, you know, it would show that it was valid. And also, the transparency about that would also give people the information about whether or not they would want to continue to come into work if there was a huge spike in positive tests. I think I would want to know, because I don’t want to continue, I wouldn't want to continue to come into*

	data being used and will help prevent or minimise other pandemics in the future.”<br><br>*“I think if the data is beneficial to understanding COVID transmission and infection rates then it should be kept and used to inform scientific and public health research (anonymised). I question whether the data would be needed for 20 years but would be happy for it to be kept.”*	*work if that was the case. I think it's important to be able to see those things and to feel safe, otherwise it's a black box and you've no idea what's going in it.”*<br><br>*“I think people probably should be told what the infection rate is in the building because, you know, ultimately if I really felt worried about it and my manager was being very unsupportive then I could still take leave if I really felt strongly.”*
**8. Communication**	Aspects of communication that would help with trust in the programme:<br><br>*“A dedicated route for enquiries.”*<br><br>*“A clear point of contact for queries or concerns rather than a 'team'.”*<br><br>*“Clear information about who manages the program internally, and who receives what information about positive tests.”*<br><br>Further information about the programme that participants suggested should be available:<br><br>*“How long the programme will run for and/or the conditions for stopping the testing.”*<br><br>*“If the sample is used for any purpose other than C-19 testing.”*<br><br>*“If there is any impact, or not, on NHS testing capacity by doing this workplace program.”*<br><br>*“What are the repercussions if you fail to take a test.”*<br><br>*“Updates on how many people test positive should only happen over a certain threshold*	How to communicate about the testing programme and about what aspects:<br><br>*“I think there needs to be a variety of ways [of communicating concerns]. They could raise it with their line manager, who then escalates, compiles a feedback and escalates to a more senior party if necessary. Probably some anonymous forums, so surveys and things, as well as maybe an email inbox where you can send in your concerns, depending on if you want your name attributed or not."*<br><br>*“I think it will be the rationale of the decision why it was introduced, why it was seen to be a mandatory rather than an optional thing. And then the reasons why they think that’s important. And also the statistics of the false positive and the false negative rates. And I think how many asymptomatic cases they might reasonably expect by doing the screening.”*

	otherwise those people could be identified by implication."<br><br>*"How often I should be tested."*<br><br>*"Who is running the test."*<br><br>*"Would like to see data on how effective asymptomatic screening is likely to be on reducing transmission."*<br><br>*"Transparency about results is of paramount importance - our health and risk levels around returning to the workplace is why I am supporting asymptomatic testing."*	
